# Farrerol inhibits proliferation and migration of colorectal cancer via the VEGF signaling pathway: evidence from network pharmacology, molecular docking, molecular dynamics simulation, and *in vitro* experiments

**DOI:** 10.3389/fphar.2025.1717293

**Published:** 2025-12-03

**Authors:** Longhui Zhang, Qijing Xu, Guanduo Sun, Xiaokang Zhang, Jialong Xue, Chun Yao, Dechun Liu, Jingming Zhai

**Affiliations:** 1 The First Affiliated Hospital, and College of Clinical Medicine of Henan University of Science and Technology, Luoyang, China; 2 The Third Affiliated Hospital of Qiqihar Medical University, Qiqihar, China

**Keywords:** network pharmacology, molecular docking, farrerol, colorectal cancer, molecular mechanism

## Abstract

**Objective:**

Although farrerol exhibits promising antitumor properties against various cancers, its potential therapeutic effects on colorectal cancer (CRC) remain unexplored, and the underlying mechanisms are still unclear. Based on network pharmacology, molecular docking, molecular dynamics simulations, and *in vitro* experiments, this study aims to investigate the molecular mechanisms of farrerol in the treatment of CRC, thereby providing new research directions for CRC therapy.

**Methods:**

This study employed network pharmacology to screen for potential therapeutic targets and pathways of farrerol in CRC, followed by preliminary validation of target validity through molecular docking and molecular dynamics simulations. Finally, *in vitro* experiments were conducted to verify the antitumor effects of farrerol against CRC.

**Results:**

Network pharmacology identified 12 key targets: CCNA1, CCNA2, CCNE1, CDC25B, CDK2, CYP19A1, ESR1, ESR2, HSP90AA1, PTPN1, RAF1, and SRC. The molecular docking results revealed that the binding energies of farrerol with all target proteins were as follows: farrerol-CCNA1 (−8.6 kcal·mol^-1^), farrerol-CCNA2 (−7.0 kcal·mol^-1^), farrerol-CCNE1 (−7.4 kcal·mol^1^), farrerol-CDC25B (−7.3 kcal·mol^-1^), farrerol-CDK2 (−10.1 kcal·mol^-1^), farrerol-CYP19A1 (−8.4 kcal·mol^-1^), farrerol-ESR1 (−3.3 kcal·mol^-1^), farrerol-ESR2 (−8.9 kcal·mol^-1^), farrerol-HSP90AA1 (−10.4 kcal·mol^-1^), farrerol-PTPN1 (−7.6 kcal·mol^-1^), farrerol-RAF1 (−4.5 kcal·mol^-1^), farrerol-SRC (−9.9 kcal·mol^-1^), farrerol-VEGFA (−8.5 kcal·mol^-1^), and farrerol-KDR (−9.0 kcal·mol^-1^). These data indicate that farrerol can spontaneously bind to the target proteins. Molecular dynamics simulations demonstrated favorable interactions within the KDR-Farrerol and VEGFA-Farrerol complexes. *In vitro* experimental results demonstrated that farrerol could inhibit the proliferation and migration of CRC cells, induce cell cycle arrest at the G0/G1 phase, and suppress the protein expression of VEGFA, VEGFR2, and p-VEGFR2.

**Conclusion:**

This study, for the first time, validated the antitumor effect of farrerol against CRC through network pharmacology, molecular docking, molecular dynamics simulations, and *in vitro* experiments. The findings indicate that the ability of farrerol to inhibit the proliferation and migration of colorectal cancer cells may be associated with the induction of G0/G1 phase cell cycle arrest and the regulation of VEGF signaling pathway activation via binding to VEGFA and KDR.

## 1 Introduction

CRC is the third most common malignant tumor worldwide, and its pathogenesis involves multiple mechanisms, including genetic susceptibility, environmental factors, and dysregulation of the immune microenvironment. Hereditary syndromes such as Lynch syndrome and familial adenomatous polyposis are well-established genetic predisposing factors, while environmental factors including high-fat diet, obesity, and chronic inflammatory states also significantly increase the risk of CRC development. Among these, chronic inflammation and dysregulated immune responses within the colorectal microenvironment play a critical role in CRC initiation and progression. Toll-like receptors (TLRs) have been identified as key mediators of intestinal inflammatory pathways, among which TLR4 promotes cell proliferation, inhibits apoptosis, and contributes to immune evasion in CRC (Jin et al., 2025; Sameer and Nissar, 2021). Over the past few decades, there has been a global increase in the incidence of early-onset CRC for no apparent reason (Patel et al., 2022). Each year, approximately 10% of all newly diagnosed cancer cases and cancer-related deaths worldwide are attributed to CRC (Bray et al., 2018). The global incidence of CRC projected to increase to 2-5 million new cases by 2035 (Arnold et al., 2017). The primary treatment modalities currently used for CRC include surgery, radiotherapy, immunotherapy, chemotherapy, and targeted therapy. These treatments have significantly alleviated symptoms, prolonged survival and improved quality of life (Rothwell et al., 2022). However, commonly used chemotherapy drugs in anticancer treatment regimens for CRC, such as 5-fluorouracil, oxaliplatin, tegafur, and cetuximab, cause varying degrees of irreversible damage to normal tissues and organs. This leads to severe adverse reactions and toxic side effects (Chen et al., 2020). For example, with respect to adagrasib combination therapy, all patients experienced treatment-related adverse events (TRAEs), with a grade 3–4 event rate of 27.7% (Yaeger et al., 2024). Additionally, drug resistance has become one of the primary challenges that clinicians must address. Therefore, developing novel, highly effective and low-toxicity CRC treatments is crucial for improving patient prognosis in the future.

Farrerol, a naturally occurring flavone compound isolated from the traditional Chinese medicine (TCM) Rhododendron mariesii Hemsl. & E.H. Wilson, has been widely used in China for its expectorant and cough-suppressing properties in the treatment of bronchitis and asthma (Qin et al., 2022). Beyond its conventional respiratory applications, farrerol exhibits a broad spectrum of pharmacological activities, including anti-inflammatory (Ci et al., 2012), oxidation (Ma et al., 2019), cardiovascular disease (Li et al., 2013), bacterial infections (Meragelman et al., 2005). More recently, its potential as an anticancer agent has garnered increasing attention. Studies have demonstrated that farrerol exerts antitumor effects in multiple cancer types through diverse mechanisms. In gastric cancer, farrerol can induce apoptosis of human gastric cancer SGC-7901 cells by activating the mitochondrial apoptosis pathway (Liu et al., 2015) and can inhibit human gastric cancer SGC-7901 cells proliferation by promoting G0/G1 cell cycle arrest (Liu et al., 2016). In hepatocellular carcinoma, farrerol inhibits transforming growth factor-β (TGF-β)-induced migration, invasion, and epithelial-mesenchymal transition (EMT) in hepatocellular carcinoma cells, and suppresses TGF-β-induced phosphorylation of Smad2/3 (Hao et al., 2024). Furthermore, farrerol induces ERK/MAPK-mediated apoptosis, attenuates TNF-mediated lipolysis, and promotes adipocyte differentiation in the ovarian cancer SKOV3 cells (Chae et al., 2021), suppresses migration, invasion, and induced the apoptosis of laryngeal squamous cell carcinoma cells via the mitochondria-mediated pathway (Zhang et al., 2021), and impedes metastatic potential in squamous cell carcinoma of the lung by regulating the expression of EMT proteins (Li et al., 2019). Additionally, farrerol has been found to upregulate pro-apoptotic molecules (Bak, Bid, cleaved caspase-3 and cleaved caspase-9) in lung adenocarcinoma, while downregulating the expression of pro-apoptotic genes (Bcl-2 and Bcl-XL) and cell cycle-related genes (CyclinD1 and CDK4) (Guo et al., 2022). Despite these promising findings across various malignancies, no existing studies have yet explored its role in the treatment of CRC. The potential therapeutic efficacy and mechanisms of farrerol in CRC remain unclear.

TCM encompasses ancient Chinese medical practices. In recent years, as people have become more aware of TCM and herbal medicines, the search for therapeutic drugs from traditional herbal formulas, and the extraction of active components from these formulas, has gradually become a global research hotspot. In the field of CRC treatment, for example, numerous drugs, including curcumin, berberine, paclitaxel, artemisinin and silymarin, have already entered clinical trials (Hao et al., 2023). This phenomenon undoubtedly underscores the reproducibility and translational potential of the anticancer effects of TCM active compounds, which have attracted significant attention from clinical researchers.

On the other hand, TCM has multiple targets and pathways for treating diseases. Therefore, it is necessary to explore the targets and pathways related to farrerol and CRC in depth. In 2007, the British pharmacologist Hopkins AL first proposed the concept of ‘network pharmacology’, defining it as a branch of pharmacology that uses network methods to analyze the ‘multi-component, multi-target, multi-pathway’ synergistic relationships between drugs, diseases, and targets (Hopkins, 2007). Based on data from systems biology, network pharmacology integrates three aspects-targets, drugs, and diseases-to extract valuable information through biological complex network analysis and validate experimental results. Its scientific and reasonable application in TCM research can enrich the traditional content of TCM and promote its modernization (Daifeng et al., 2024). Therefore, we can use network pharmacology methods to analyze the role of farrerol in CRC.

This study screened and predicted potential targets and signaling pathways for farrerol treatment of CRC using a database and then used network pharmacology methods to study the relationships among drugs, targets, and pathways. Molecular docking was used to study the interaction between farrerol and CRC treatment targets. These findings provide a scientific basis for later drug development and clinical application.

## 2 Materials and methods

### 2.1 Database and research process

**FIGURE 1:**
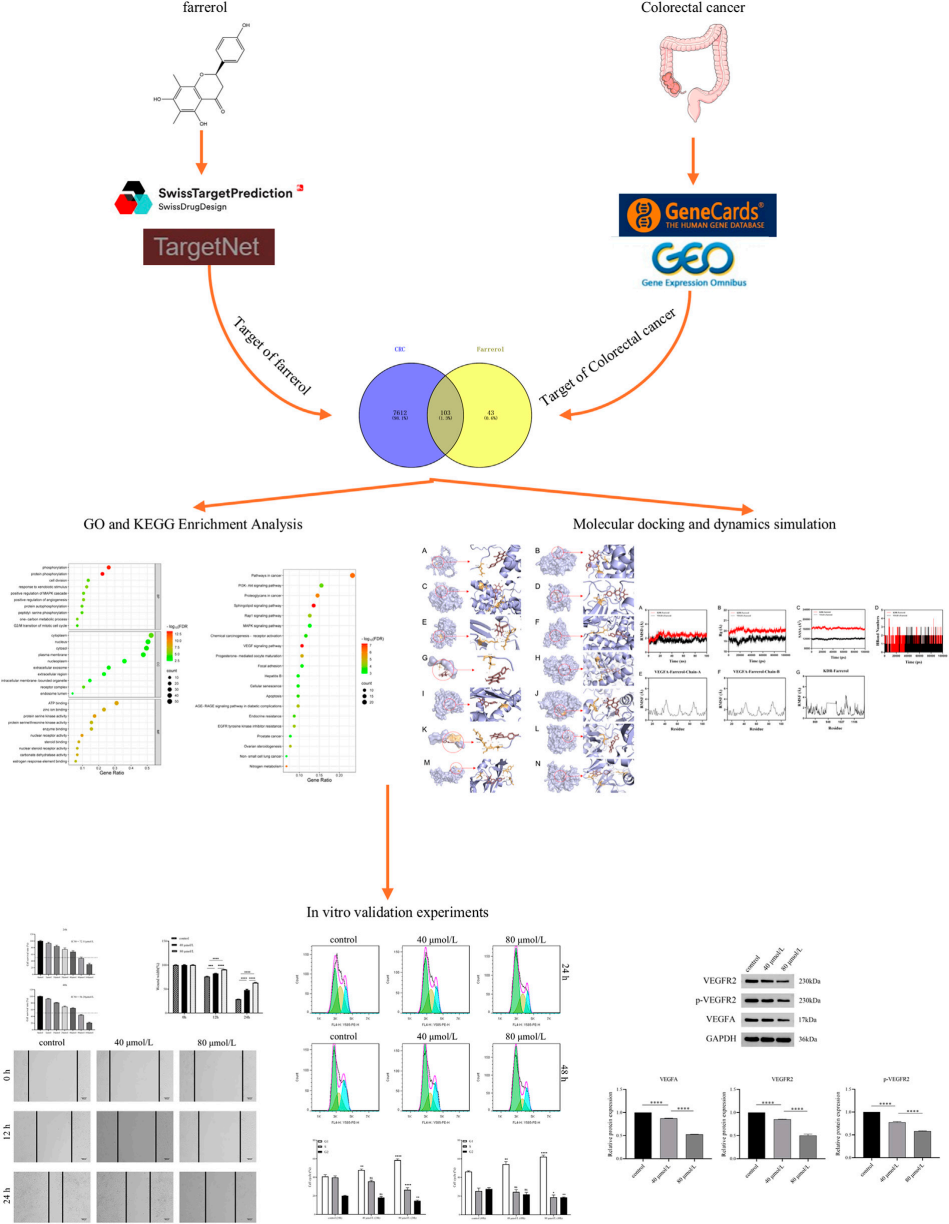
This study is a detailed flow chart.

The databases involved in this study (Table 1) and the research process outline (Figure 1).

**TABLE 1:** Basic information of the database used for the screening of farrerol in the treatment of colorectal cancer.

Name	URL
PubChem	https://pubchem.ncbi.nlm.nih.gov/
TargetNet	http://targetnet.scbdd.com/
SwissTargetPrediction	http://www.swisstargetprediction.ch/
GeneCards database	https://www.genecards.org/
GEO database	https://www.ncbi.nlm.nih.gov/geo/
UniProt	https://www.uniprot.org/
Venny 2.1.0	https://bioinfogp.cnb.csic.es/tools/venny/index.html
STRING	https://cn.string-db.org/
DAVID	https://davidbioinformatics.nih.gov/
Bioinformatics	https://www.bioinformatics.com.cn/
RCSB PDB	https://www.rcsb.org/
GEPIA	http://gepia.cancer-pku.cn/
HPA	https://www.proteinatlas.org/
KEGG mapper	https://www.kegg.jp/kegg/mapper/
AlphaFold	https://alphafold.ebi.ac.uk/

### 2.2 Access to potential targets of farrerol

The PubChem (https://pubchem.ncbi.nlm.nih.gov/) database was utilized to search for details of farrerol (Kim et al., 2025). The relevant targets of farrerol were predicted via farrerol’s SDF file or farrerol’s canonical SMILES in SwissTargetPrediction (http://www.swisstargetprediction.ch/), TargetNet (http://targetnet.scbdd.com/) (Daina et al., 2019; Yao et al., 2016). The screening threshold was set at Probability >0. The obtained targets were translated into gene names via the UniProt database (https://www.uniprot.org/) (UniProt, 2025). The obtained target genes were subsequently combined and de-duplicated. The results revealed the potential targets of farrerol.

### 2.3 CRC-related target collection

The genes associated with CRC were obtained from the GeneCards Database (https://www.genecards.org/) (Stelzer et al., 2016) using the keyword “colorectal cancer”. Genes related to CRC obtained from the GeneCards database were filtered to include those above the median value. GSE44076 was selected from the GEO database (https://www.ncbi.nlm.nih.gov/geo/) as the study subject (Clough and Barrett, 2016). The screening of differentially expressed genes was conducted via the GEO2R tool, with the screening criterion set at adj.P.Val <0.05 and |logFC| > 1. The obtained differentially expressed genes (DEGs) obtained via TBtools (Chen et al., 2023) after the removal of duplicates are presented in volcano plots. The DEGs obtained from the GSE44076 dataset were subsequently intersected with CRC-related targets obtained from the GeneCards database to obtain CRC disease-related targets.

### 2.4 Drug-disease common target screening and PPI network construction

The intersection of farrerol targets and CRC targets was determined via Venny 2.1.0 (https://bioinfogp.cnb.csic.es/tools/venny/index.html) and the intersected genes represented the potential targets of farrerol for CRC treatment. The potential interaction targets were imported into the String database 12.0 (https://cn.string-db.org/) (Szklarczyk et al., 2023) with the species set as *Homo sapiens*, the minimum required interaction score as the highest confidence of 0.9, and the network display options were limited to hiding disconnected nodes in the network to obtain the PPI relationships. The tsv file was downloaded and imported into Cytoscape (version 3.10.2) (Shannon et al., 2003) for visualization.

### 2.5 GO and KEGG enrichment analysis

GO and KEGG enrichment analyses of potential interaction targets of farrerol for CRC treatment were performed via the Functional Annotation Tool on the DAVID website (https://davidbioinformatics.nih.gov/) (Sherman et al., 2022). The organization and visualization of the obtained data was performed via bioinformatics (https://www.bioinformatics.com.cn/) (Tang et al., 2023).

### 2.6 Drug-target-pathway network construction

The potential targets of farrerol in CRC, farrerol and KEGG pathways were imported into Cytoscape for visualization and analysis of their relationships. The nodes represent farrerol, genes or pathways, and the connecting lines represent the relationships among biomolecules.

### 2.7 Screening core targets

The PPI’s tsv file was imported into Cytohubba of Cytoscape (version 3.10.2), and degree, maximum neighbourhood component (MNC), maximal clique centrality (MCC) and closeness were used to filter the top 20 targets. The intersection of the targets obtained via these four calculation methods is the core target.

### 2.8 Molecular docking

The SDF of farrerol was downloaded from PubChem, and Chem3D (version 20.0.0.41) was utilized to optimize the molecular structure by minimizing energy. The resulting file was saved in mol2 format. The mol2 format of the ligand was imported into AutoDockTools (version 1.5.7) and exported as a pdbqt format file. The PDB files and details of the target proteins were obtained from the PDB database (https://www.rcsb.org/) or AlphaFold database (https://alphafold.ebi.ac.uk/) (Berman et al., 2000; Tunyasuvunakool et al., 2021) (Table 2). PyMOL (version 3.1.1) was utilized to process the receptor protein files to remove water molecules, ligands and ions. Proteins were hydrogenated in AutoDockTools and output as a file in pdbqt format. The pdbqt files of the receptor and ligand were imported into AutoDockTools. When the docking box was constructed, the receptor protein was centered, the docking box was positioned to cover the receptor protein entirely, and the ligand was located outside the docking box. The parameters of the docking box were collected (Table 3). Molecular docking was performed in AutoDock Vina (version 1.1.2) (Trott and Olson, 2010) and repeated three times for each target to select the optimal conformation based on the binding energy. When the binding energy was the same for all three repetitions, the best result was obtained on the basis of the root mean square deviation (RMSD). The magnitude of the binding energy reflects the possibility of binding between the receptor and ligand. As the binding energy decreases, the receptor and ligand exhibit an increased degree of affinity, and the conformations of the receptor and ligand become more stable. Finally, the molecular docking results were visualized via PyMOL.

**TABLE 2:** Details of the protein targets in the PDB database.

Targets	PDB/AlphaFold ID	Method	Resolution (Å)	R-Value free	R-Value work	R-Value observed
CCNA1	P78396-F1-v6	AlphaFold	—	—	—	—
CCNA2	7ACK	X-RAY DIFFRACTION	1.800	0.232	0.197	0.197
CCNE1	8VQ3	X-RAY DIFFRACTION	1.840	0.217	0.199	0.200
CDC25B	4WH9	X-RAY DIFFRACTION	1.500	0.152	0.126	0.127
CDK2	7RXO	X-RAY DIFFRACTION	1.380	0.233	0.220	0.221
CYP19A1	5JKV	X-RAY DIFFRACTION	2.750	0.236	0.214	0.215
ESR1	8AFN	X-RAY DIFFRACTION	1.360	0.185	0.173	0.174
ESR2	7XVY	X-RAY DIFFRACTION	1.540	0.237	0.206	—
HSP90AA1	8SBT	X-RAY DIFFRACTION	1.500	0.197	0.173	0.174
PTPN1	7KLX	X-RAY DIFFRACTION	1.840	0.205	0.196	0.197
RAF1	8A6F	X-RAY DIFFRACTION	1.600	0.181	0.162	0.163
SRC	6E6E	X-RAY DIFFRACTION	2.150	0.285	0.250	0.252
VEGFA	6BFT	X-RAY DIFFRACTION	2.550	0.220	0.170	0.173
KDR (VEGFR2)	3WZE	X-RAY DIFFRACTION	1.900	0.224	0.185	0.188

**TABLE 3:** Grid docking parameters for molecular docking.

Target name	PDB/AlphaFold ID	Spacing (angstrom)	Center grid box			Number of grid points			Exhaustiveness
			X center	Y center	Z center	X	Y	Z	
CCNA1	P78396-F1-v6	1	−10.733	1.127	−0.495	40	40	40	32
CCNA2	7ACK	1	−19.936	2.998	−1.031	54	54	54	32
CCNE1	8VQ3	1	29.856	−9.997	−22.613	50	50	50	32
CDC25B	4WH9	1	5.669	−11.259	−10.281	50	50	50	32
CDK2	7RXO	1	−10.186	4.806	−12.934	60	60	60	32
CYP19A1	5JKV	1	83.344	50.061	46.451	74	74	74	32
ESR1	8AFN	1	15.425	12.918	11.285	8	12	10	32
ESR2	7XVY	1	−8.158	10.549	−25.32	58	48	64	32
HSP90AA1	8SBT	1	−0.212	15.079	20.232	44	42	42	32
PTPN1	7KLX	1	42.808	16.246	15.165	64	44	40	32
RAF1	8A6F	1	25.606	12.987	−7.795	14	14	32	32
SRC	6E6E	1	27.389	−3.776	16.621	38	46	58	32
VEGFA	6BFT	1	−43.816	0.863	128.735	64	40	42	32
KDR	3WZE	1	19.637	23.919	29.937	48	50	54	32

Since the experimental crystal structure of CCNA1 was not available in the PDB, the CCNA1 structure predicted by AlphaFold2 (AF-P78396-F1-v6) was selected, with an average pLDDT score of 67.62 (51.8% very high, 3.4% high, 2.6% low, and 42.2% very low). Most of the low and very low confidence regions were located at the protein termini. Hydrogen atoms were added under physiological pH using the PyMOL software, and energy minimization was performed on the entire protein using the Optimize plugin. The optimization procedure employed the GAFF force field and the conjugate gradient algorithm, with 5,000 steps performed while keeping the protein backbone atoms fixed to preserve the integrity of the core structure. During grid generation for molecular docking, the docking box was ensured to fully cover the entire protein.

### 2.9 Molecular dynamics simulation

This study employed molecular dynamics simulations using Gromacs 2022.2. Force field parameters were obtained using the pdb2gmx tool in Gromacs and the AutoFF website. During the simulation, the molecular parameters of the receptor protein were based on the CHARMM36 (Jo et al., 2008) force field, while those of the ligand were based on the CGenff force field. A 1 nm TIP3P-type cubic water box was added around the system for solventization (Mark and Nilsson, 2001). Ions were added to the system using the gmx genion tool to achieve electrical neutrality. Long-range electrostatic interactions were handled using the Particle Mesh Ewald (PME) method, with a cutoff distance of 1 nm. All bonds were constrained using the SHAKE algorithm, and the molecular dynamics simulation was performed using the leap-frog integrator with Verlet neighbor list with an integration step size of 1 fs. The system was energy-optimized prior to the molecular dynamics simulation. The energy minimization process included 3,000 steps of steepest descent optimization followed by 2,000 steps of conjugate gradient optimization. The optimization steps are as follows: first, the solute is constrained, and the water molecules are minimized; then, the counterions are constrained, and the system is minimized; finally, the entire system is minimized without constraints. The simulation was run under NPT conditions at a temperature of 310 K and constant pressure, with a simulation time of 100 ns. During the simulation, the gmx rms, gmx rmsf, gmx hbond, gmx Rg, and gmx sasa tools were used to calculate the root mean square deviation (RMSD), root mean square fluctuation (RMSF), hydrogen bonds (HBonds), radius of gyration (Rg), and solvent accessible surface area (SASA), respectively. The binding free energy of the complex was calculated using the gmx_MMPBSA1.6.4 package in GROMACS. Trajectory segment length (last 20 ns), frame interval (every 100 ps), internal dielectric constant (1), external dielectric constant (80), surface model (LCPO), ionic strength (0.15 M), and probe radius (1.4 Å).

### 2.10 Reagents

Fetal bovine serum (catalog number. FSP500) from Excell; 1,640 medium (catalog number. iCell-0012) from iCell Bioscience Inc. (catalog number. FSP500); PBS (catalog number. G4202) and Cell Counting Kit-8 (catalog number. G4103) from Wuhan Servicebio Technology Co., Ltd.; Propidium Iodide (catalog number. CA1020) purchased from Beijing Solarbio Science & Technology Co., Ltd. BCA protein quantification kit (catalog number. CBW0020), SDS-PAGE gel preparation kit (catalog number. CBW0001-CBW0010), and farrerol (catalog number. CB-0158) were purchased from Wuhan Kebei Technology Co., Ltd. Anti-GAPDH (catalog number. 60004-1-Ig) was purchased from Proteintech Group, Inc. VEGFA (#2478), VEGFR2 (#2479), and p-VEGFR2 (#50661) were purchased from Cell Signaling Technology.

### 2.11 Cell cuture and treatment

HCT116 cells were provided by Wuhan Pricella Biotechnology Co., Ltd. and cultured in 1,640 medium supplemented with 10% fetal bovine serum to ensure optimal growth conditions. The cells were maintained in an incubator at a constant temperature of 37 °C with 5% CO_2_. The cell experiments were divided into three groups: control group, low-dose farrerol group, and high-dose farrerol group.

### 2.12 Cell counting Kit-8 (CCK-8) assay

HCT116 cells were seeded in 96-well plates at a density of 1*10^4^ cells per well in 100 μL of culture medium. The plates were incubated at 37 °C under 5% CO_2_ until the cells adhered and reached normal growth. Cells were then treated according to the experimental groups. Farrerol was administered at concentrations ranging from 0 to 160 μmol/L, using 2-fold serial dilutions, for 24 and 48 h, respectively. Subsequently, 10 μL of CCK-8 solution was added to each well. Wells containing the corresponding volume of culture medium and CCK-8 solution without cells were used as blank controls. The plates were further incubated in the cell culture incubator for 1 h, and the absorbance was measured at 450 nm using a microplate reader. The half maximal inhibitory concentration (IC50) was calculated based on the results, and the concentrations for subsequent experiments were selected according to the IC50 values.

### 2.13 Wound healing assay

Cells in the logarithmic growth phase were seeded into 6-well plates at a density of 5*10^5^ cells/mL and incubated in a cell culture incubator. After the cells reached confluence, a scratch wound was created on the cell monolayer. The cells were then washed with PBS to remove detached cells. According to the experimental grouping, corresponding drugs were added, and the plates were returned to the incubator for continued culture. Serum-free medium was used without the addition of mitomycin C. Images were captured at 0, 12, and 24 h. Analysis was conducted using Photoshop and ImageJ software. Wound width was calculated as wound area divided by wound height. Each experiment was performed in triplicate.

### 2.14 Flow cytometry

Cells in the logarithmic growth phase were seeded into 6-well plates at a density of 5*10^6^ cells per well and cultured in an incubator until adherent. After 24 h and 48 h of drug treatment, the cells were digested with trypsin and collected. After fixation with 70% ethanol, a permeabilization solution (100 μg/mL RNase A, 0.2% Triton X-100) was added and incubated at 37 °C for 30 min. Finally, staining was performed with 50 μg/mL propidium iodide for 10 min. Samples were analyzed by flow cytometry, and data analysis was conducted using FlowJo v7.6.1. The analysis included event counting (10,000 events per sample) and gating strategy: FSC-H vs. SSC-H was used to exclude debris and dead cells, selecting the intact cell population; FL4-H (Y585-PE-H) vs. SSC-H was further applied to identify the target cell population. All experiments were performed in triplicate.

### 2.15 Western blot

Total protein was extracted from the harvested and processed cells. The protein concentration of each sample was determined using a BCA assay kit. Proteins were separated by SDS-PAGE and subsequently transferred onto a polyvinylidene fluoride (PVDF) membrane. The membrane was blocked with 3% bovine serum albumin for 30 min, followed by incubation with diluted primary antibodies at 4 °C for 15 h. Thereafter, the membrane was incubated with secondary antibodies at room temperature for 30 min. The PVDF membrane was then placed in a developing solution and visualized in a darkroom. Quantification of the grayscale values for the target bands and the internal reference GAPDH bands was performed using AlphaEase FC software. The relative expression level of the target protein was calculated as the ratio of its grayscale value to that of the corresponding GAPDH band, in order to correct for variations in loading amounts. Furthermore, during the experimental process, samples from both the experimental and control groups were run simultaneously on the same gel to minimize inter-gel variability.

### 2.16 Statistical analysis

All data are presented as the mean ± standard deviation (SD) from at least three independent experiments. For comparisons between two groups: data conforming to a normal distribution and homogeneity of variance were analyzed using a two-tailed unpaired Student’s t-test; otherwise, the Mann-Whitney U test was applied. For comparisons among three or more groups, one-way ANOVA was used. If the ANOVA indicated a significant difference, Tukey’s test was subsequently employed for all pairwise comparisons. Prior to parametric tests (t-test, ANOVA), the normality of the data distribution was assessed using the Shapiro-Wilk test. For multiple group comparisons, P-values were adjusted using the aforementioned Tukey’s test method. All statistical analyses and graph generation were performed using GraphPad Prism software (version 8.0.0). All ‘n' values in the text represent the number of biological replicates. Multiple measurements within a single biological replicate (e.g., technical replicates) were averaged prior to analysis, and this mean value was treated as one independent ‘n' value for the final statistical analysis. A p value <0.05 was considered statistically significant.

## 3 Results

### 3.1 Targets of farrerol and CRC

**FIGURE 2:**
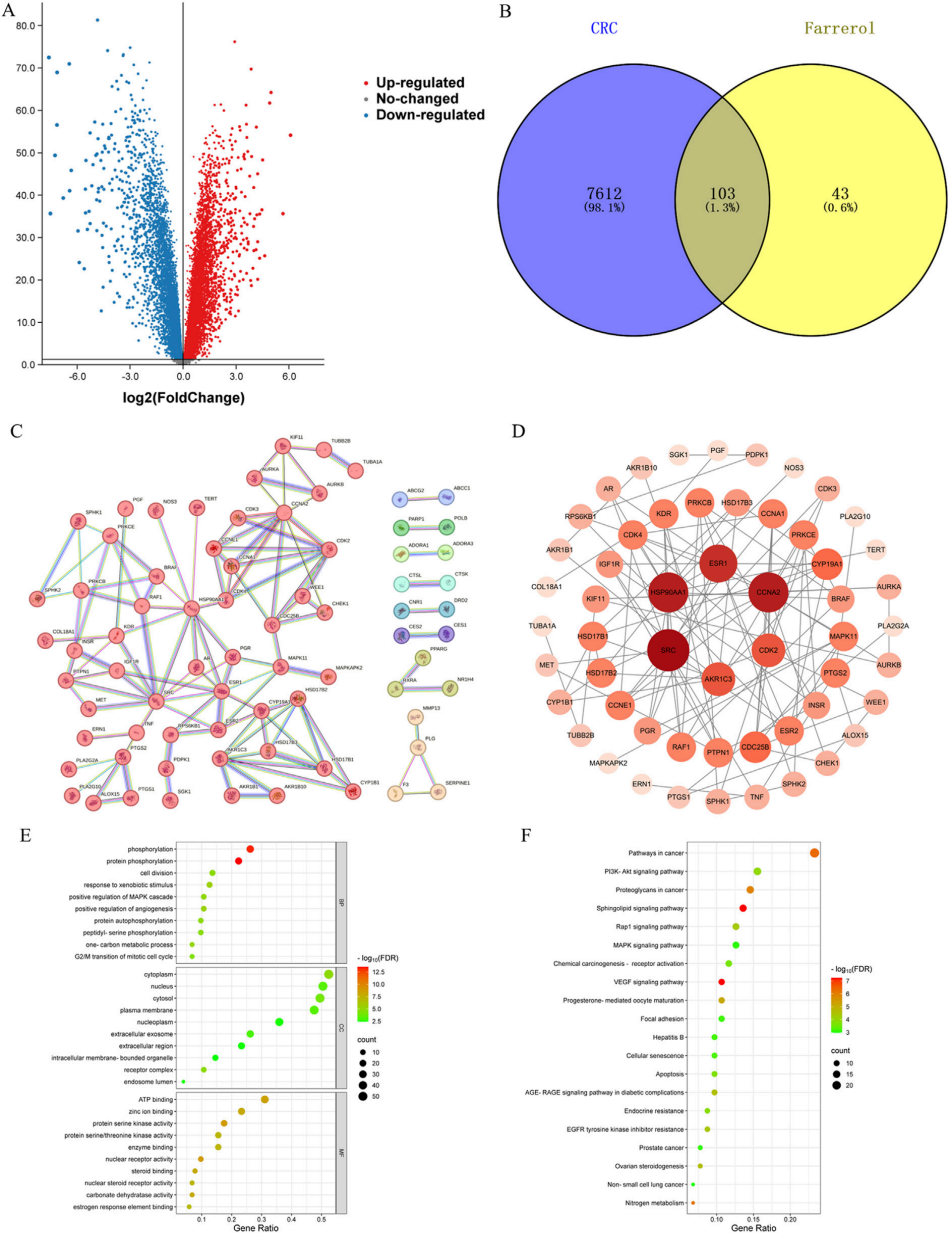
**(A)** Volcano plot of DEGs associated with CRC. **(B)** Venny diagram showing the common part of farrerol and CRC. **(C)** PPI network of potential targets for farrerol therapy of CRC. **(D)** The PPI network data were visualized via Cytoscape. **(E)** GO functional enrichment analysis of farrerol in CRC. **(F)** KEGG pathway enrichment analysis of farrerol in CRC.

A total of 146 farrerol action targets were obtained from the SwissTargetPrediction and TargetNet databases. A total of 2,964 differentially expressed genes were identified in the GSE44076 dataset following screening (Figure 2A). A total of 11,840 CRC-related targets were obtained via GeneCards. The median number of CRC-related targets was determined by sorting the ‘relevance score’ in descending order, and 5920 CRC-related targets were obtained. A total of 7715 CRC-related genes were obtained by combining 2,964 differentially expressed genes and 5920 CRC-related targets.

### 3.2 Common target acquisition and PPI network construction

The results of the Venn diagram analysis revealed that a total of 103 common targets were identified by intersecting 146 drug targets with 7,715 disease targets (Figure 2B). These 103 common targets are potential targets of farrerol for the treatment of CRC. A total of 103 targets were imported into the STRING database to construct the PPI network (Figure 2C). The PPI network data were visualized via Cytoscape 3.10.2 (Figure 2D). When the nodes are larger and darker, the degree of the node is greater.

### 3.3 GO and KEGG pathway enrichment analysis

**FIGURE 3:**
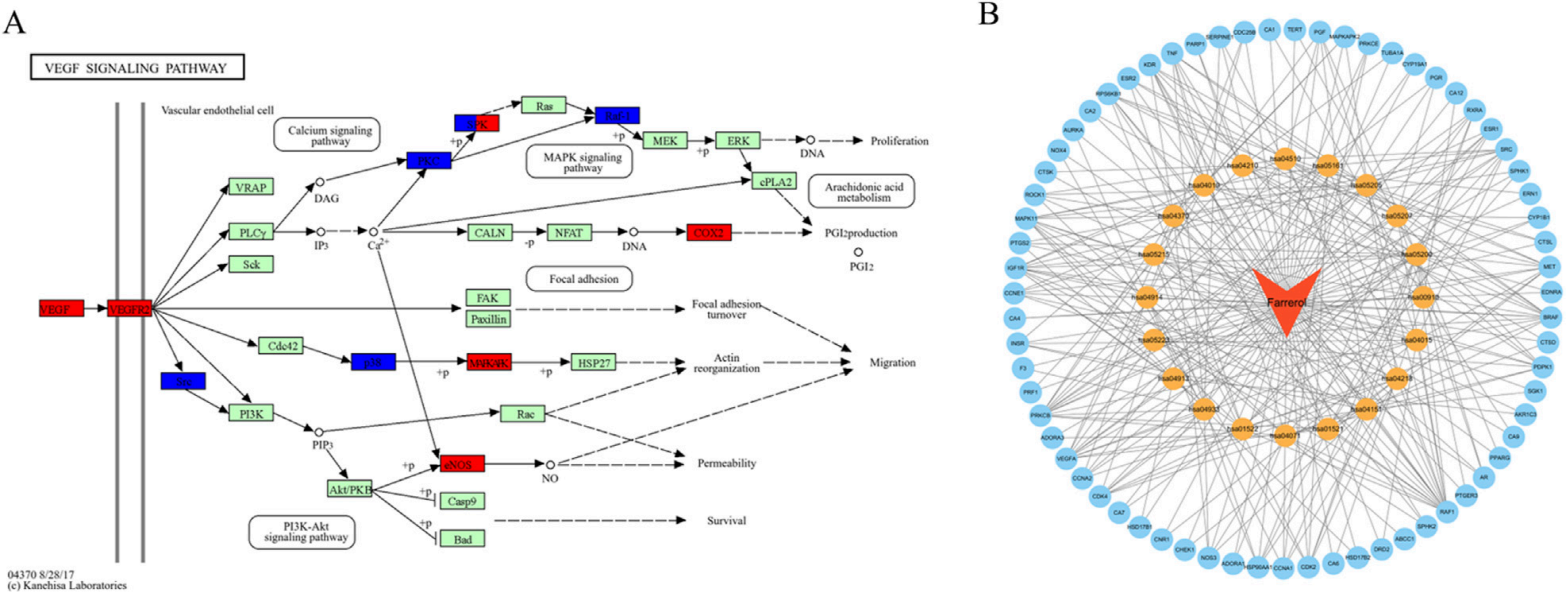
**(A)** VEGF signaling pathway (the red markers represent highly expressed genes in GSE44076, whereas the blue markers represent genes with low expression). **(B)** Drug-target-pathway network (the blue circles represent targets, the yellow circles represent pathways, and the orange triangle represents farrerol).

A total of 103 potential targets of farrerol for CRC treatment were imported into DAVID for GO and KEGG enrichment analysis. GO analysis enriched 519 terms (p < 0.01), including BP (biological progress): 352, CC (cellular component):67 and MF (molecular function):100. For each analysis type, the 10 smallest p values were selected for bioinformatics visualization (Figure 2E). The results indicate that 103 targets were involved in biological processes, such as protein phosphorylation, positive regulation of angiogenesis, one-carbon metabolic process, positive regulation of the MAPK cascade, and G2/M transition of the mitotic cell cycle, etc. The main cellular components involved were the cytoplasm, receptor complex, plasma membrane, nucleus, and extracellular exosome, etc. The main molecular functions involved were protein serine kinase activity, nuclear receptor activity, ATP binding, zinc ion binding, protein serine/threonine kinase activity, etc. KEGG pathway enrichment analysis identified 105 signaling pathways and visualized the top 20 pathways (Figure 2F), which mainly involved Pathways in cancer, PI3K-Akt signaling pathway, Proteoglycans in cancer, Sphingolipid signaling pathway, MAPK signaling pathway, Rap1 signaling pathway, etc. Analysis of the KEGG results revealed that the VEGF signaling pathway exhibited the highest fold enrichment and the smallest p value among pathways directly associated with cancer progression and angiogenesis. Therefore, the VEGF signaling pathway was considered profound and selected for further mapping (Figure 3A). The marked genes in the figure indicate potential targets for farrerol intervention.

### 3.4 Drug-target-pathway network construction

The top 20 KEGG pathways were imported into Cytoscape to construct a drug-target-pathway network (Figure 3B). These results demonstrated that farrerol exerts its effects in treating CRC through multiple targets and multiple signaling pathways.

### 3.5 Molecular docking validation of farrerol and core targets

**FIGURE 4:**
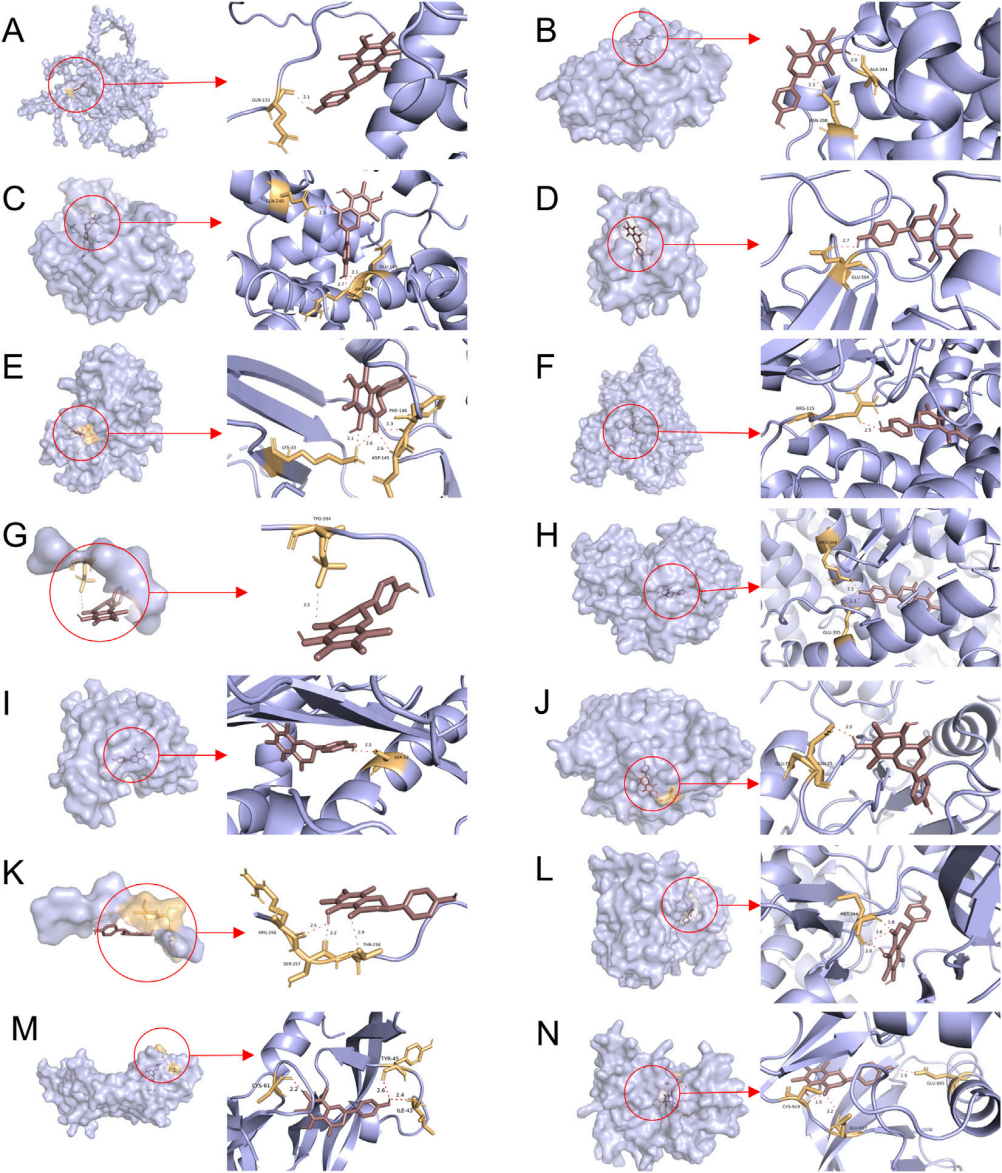
Molecular docking patterns of farrerol and core target proteins. **(A)** Farrerol-CCNA1. **(B)** Farrerol-CCNA2. **(C)** Farrerol-CCNE1. **(D)** Farrerol-CDC25B. **(E)** Farrerol-CDK2. **(F)** Farrerol-CYP19A1. **(G)** Farrerol-ESR1. **(H)** Farrerol-ESR2. **(I)** Farrerol-HSP90AA1. **(J)** Farrerol-PTPN1. **(K)** Farrerol-RAF1. **(L)** Farrerol-SRC. **(M)** Farrerol-VEGFA. **(N)** Farrerol-KDR.

The PPI network diagram of potential targets was constructed via the CytoHubba plugin. This analysis resulted in the identification of 12 core targets (Table 4). Analysis of the KEGG results revealed that the VEGF signaling pathway exhibited the highest fold enrichment and the smallest p value among pathways directly associated with cancer progression and angiogenesis. Since VEGFA and KDR are key targets within the VEGF signaling pathway, they were selected for inclusion in the molecular docking study. The molecular docking of the screened core targets and the key targets in the VEGF signaling pathway was performed in AutoDock Vina (version 1.1.2). The binding energy, amino acid residues and hydrogen bonds were collected (Table 5). However, no protein file for CCNA1 was found in the PDB database, so the CCNA1 protein data from the AlphaFold database (https://alphafold.com/) were selected (Jumper et al., 2021). The molecular docking results revealed that the binding energies of farrerol with all target proteins were as follows: farrerol-CCNA1 (−8.6 kcal·mol^-1^), farrerol-CCNA2 (−7.0 kcal·mol^-1^), farrerol-CCNE1 (−7.4 kcal·mol^1^), farrerol-CDC25B (−7.3 kcal·mol^-1^), farrerol-CDK2 (−10.1 kcal·mol^-1^), farrerol-CYP19A1 (−8.4 kcal·mol^-1^), farrerol-ESR1 (−3.3 kcal·mol^-1^), farrerol-ESR2 (−8.9 kcal·mol^-1^), farrerol-HSP90AA1 (−10.4 kcal·mol^-1^), farrerol-PTPN1 (−7.6 kcal·mol^-1^), farrerol-RAF1 (−4.5 kcal·mol^-1^), farrerol-SRC (−9.9 kcal·mol^-1^), farrerol-VEGFA (−8.5 kcal·mol^-1^), and farrerol-KDR (−9.0 kcal·mol^-1^). A binding energy of <0 kcal·mol^-1^ indicates spontaneous binding of the ligand to the receptor, whereas a binding energy of <−5 kcal·mol^-1^ indicates strong binding activity and a stable structure. These results indicate that farrerol spontaneously binds to core target proteins and strongly binds to CCNA1, CCNA2, CCNE1, CDC25B, CDK2, CYP19A1, ESR2, HSP90AA1, PTPN1, VEGFA, KDR and SRC. Farrerol may exert anticancer effects by binding to these core target proteins. The results of the molecular docking are visualized (Figure 4).

**TABLE 4:** Hub targets identified via 4 different algorithms in the Cytohubba plugin.

Gene symbol	Full name
CCNA1	Cyclin A1
CCNA2	Cyclin A2
CCNE1	Cyclin E1
CDK2	Cyclin-dependent kinase 2
CDC25B	Cell division cycle 25B
CYP19A1	Cytochrome P450 family 19 subfamily a member 1
HSP90AA1	Heat shock protein 90 alpha family a member 1
ESR1	Estrogen receptor 1
ESR2	Estrogen receptor 2
RAF1	Raf-1 proto-oncogene, serine/threonine kinase
SRC	SRC proto-oncogene, non-receptor tyrosine kinase
PTPN1	Protein tyrosine phosphatase non-receptor type 1

**TABLE 5:** Basic information on the molecular docking of farrerol and target proteins.

Targets	PDB/AlphaFold ID	Residue involved in H bonding	H-bond length (Å)	Binding energy (kcal·mol^-1^)
CCNA1	P78396-F1-v6	GLN-133	2.1	−8.60
CCNA2	7ACK	ASN-208; ALA-344	2.3; 2.0	−7.00
CCNE1	8VQ3	ARG-148; GLU-149; GLN-240	2.7; 2.1; 2.3	−7.40
CDC25B	4WH9	GLU-504	2.7	−7.30
CDK2	7RXO	LYS-33; ASP-145; PHE-146	2.1; 2.6; 2.6; 2.3	−10.10
CYP19A1	5JKV	ARG-115	2.5	−8.40
ESR1	8AFN	TPO-594	3.5	−3.30
ESR2	7XVY	GLU-305; ARG-346	2.3; 2.2	−8.90
HSP90AA1	8SBT	SER-52	2.5	−10.40
PTPN1	7KLX	GLU-75	2.5	−7.60
RAF1	8A6F	ARG-256; SER-257; THR-258	2.5; 2.2; 2.9	−4.50
SRC	6E6E	MET-344	2.0; 3.6; 1.8	−9.90
VEGFA	6BFT	ILE-43; TYR-45; CYS-61	2.4; 2.6; 2.2	−8.50
KDR	3WZE	GLU-885; GLU-917; CYS-919	2.8; 3.2; 1.9	−9.00

### 3.6 Molecular dynamics simulation

**FIGURE 5:**
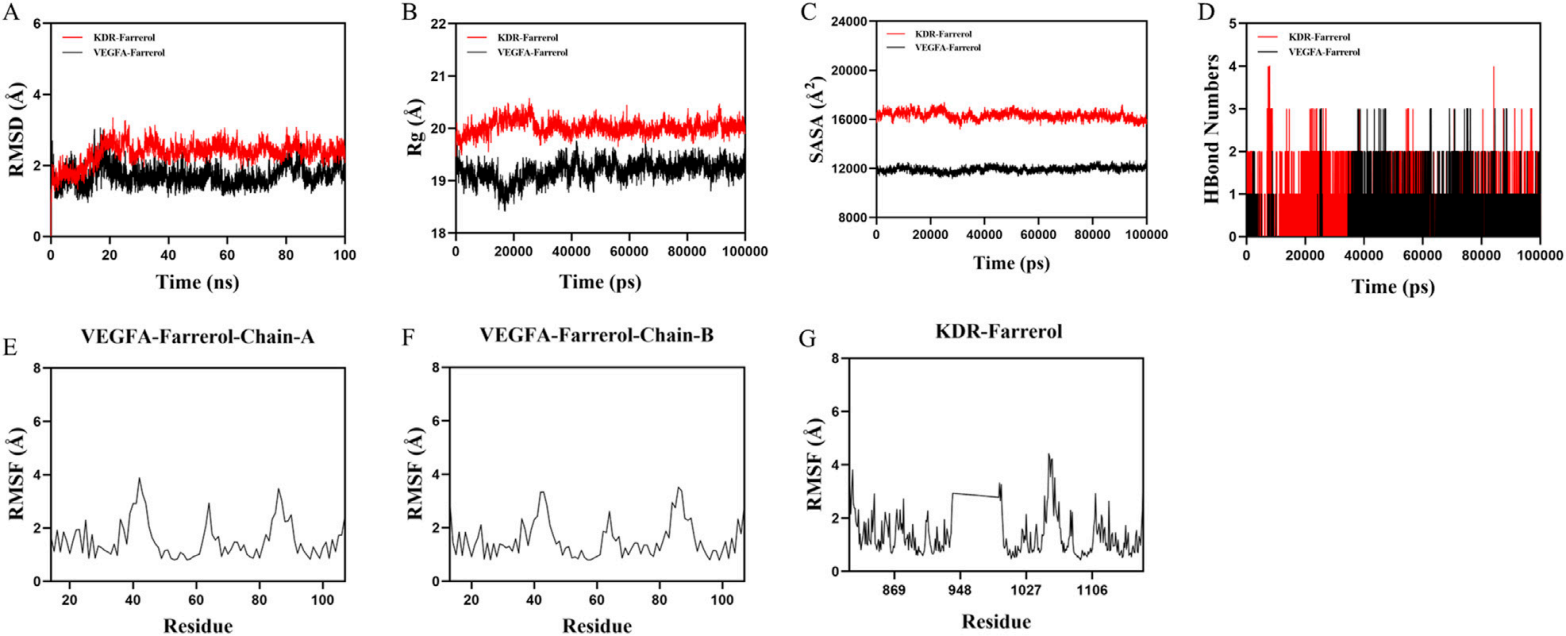
Molecular dynamics simulation of protein-ligand complexes. **(A)** RMSD values of protein-ligand complexes over time. **(B)** Rg values of protein-ligand complexes over time. **(C)** SASA values of protein-ligand complexes over time. **(D)** HBonds values of protein-ligand complexes over time. **(E–G)** RMSF values of protein-ligand complexes.

Based on the molecular docking results, VEGFA and KDR were found to rank highly in terms of binding energy and are key targets in the VEGF signaling pathway. Therefore, VEGFA and KDR were selected for molecular dynamics simulation studies. To validate the binding stability between the ligand and protein receptor, we used Gromacs 2022 to calculate the root mean square deviation (RMSD), radius of gyration (Rg), solvent accessible surface area (SASA), hydrogen bonds, and root mean square fluctuation (RMSF) of the KDR-Farrerol and VEGFA-Farrerol complexes. The RMSD results showed that the KDR-Farrerol complex reached equilibrium after 20 ns, ultimately fluctuating around 2.1 Å, while the VEGFA-Farrerol complex reached equilibrium after 25 ns, with the final value fluctuating around 1.6 Å (Figure 5A). These results indicate that Farrerol exhibits high stability when bound to KDR and VEGFA target proteins, respectively. The Rg results showed slight fluctuations in the KDR-Farrerol and VEGFA-Farrerol complex systems during motion (Figure 5B). This suggests that the small molecule-target protein complex undergoes conformational changes during motion. The SASA results also showed slight fluctuations in the KDR-Farrerol and VEGFA-Farrerol complex systems (Figure 5C). These results demonstrate that the bound small molecule affects the binding microenvironment, leading to changes in SASA.

Hydrogen bonds play an important role in ligand binding to proteins. Figure 5D shows that the number of hydrogen bonds between the KDR-Farrerol ranges from 0 to 4, with most complexes having approximately two hydrogen bonds. The number of hydrogen bonds between the VEGFA-Farrerol ranges from 0 to 3, with most complexes having approximately one hydrogen bond. These results indicate that the ligand has good hydrogen-bonding interactions with the target protein.

RMSF indicates the flexibility of amino acid residues in a protein. Figures 5E–G shows that the RMSF values of the KDR-Farrerol and VEGFA-Farrerol complexes are mostly below 3 Å, indicating lower flexibility and higher stability.

**FIGURE 6:**
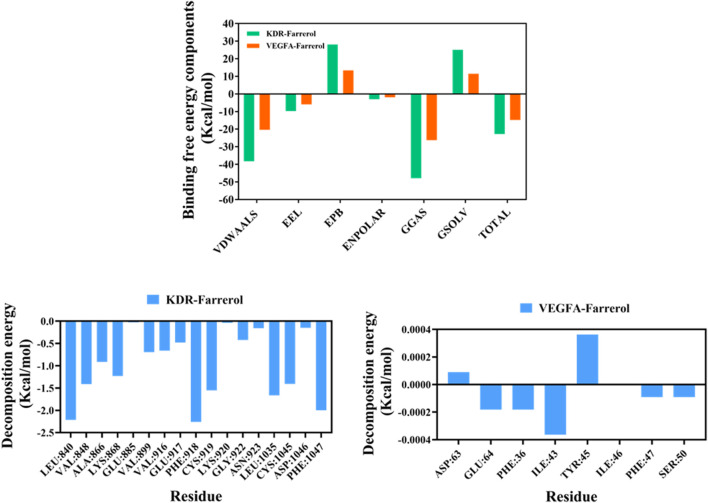
MMPBSA-based free energy calculation.

Next, we used the MM/PBSA method to calculate the binding free energy between the small molecule and the target protein (Figure 6). The binding free energies for the KDR-Farrerol and VEGFA-Farrerol complexes were −22.78 kcal·mol^-1^ and -14.77 kcal·mol^-1^, respectively. Negative values indicate binding affinity for the target protein; lower values indicate stronger binding. Subsequently, this study calculated and analyzed the amino acids that significantly contribute to small molecule binding in the complex system. In the KDR-Farrerol complex system, the results showed that residues LEU840, PHE918, PHE1047, LEU1035, CYS919, CYS1045, VAL848, and LYS868 had high contribution values (Figure 6). In the VEGFA-Farrerol complex, the residues ILE43, PHE36, GLU64, PHE47, and SER50 had higher contribution values (Figure 6). These results suggest that these amino acid residues may play an important role in the catalytic process.

### 3.7 Farrerol inhibits the proliferation of CRC cells

**FIGURE 7:**
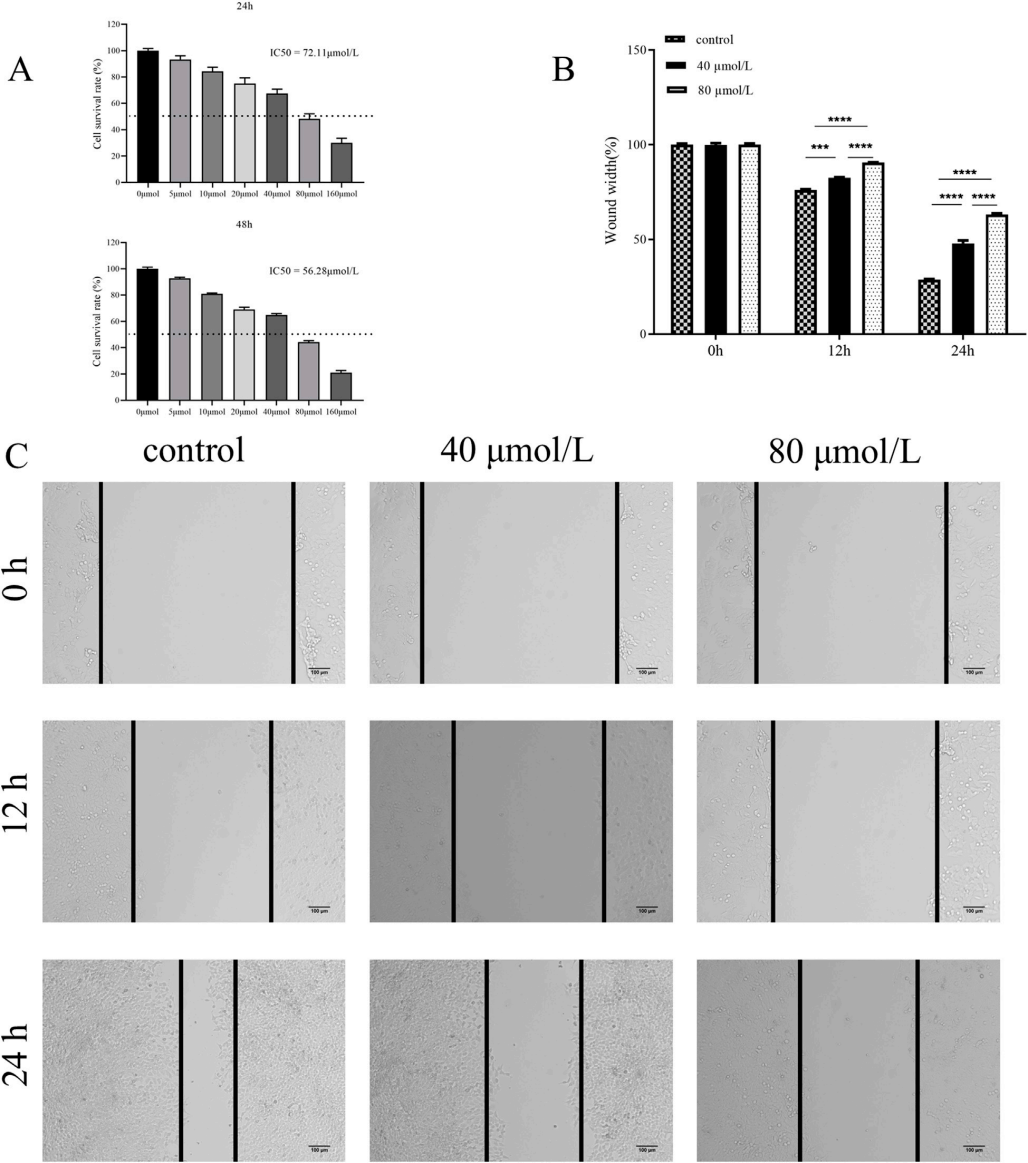
Farrerol inhibits proliferation and migration of CRC Cells. **(A)** HCT116 cells were treated with various concentrations of farrerol for 24 and 48 h,and cell proliferation was determined using a CCK-8 assay. **(B,C)** The migratory ability of HCT116 cells was assessed by a wound healing assay after treatment with 40 μmol/L (low) and 80 μmol/L (high) farrerol for 0, 12, and 24 h. Data are presented as the mean ± standard deviation from three independent experiments. *p < 0.05, **p < 0.01, ***p < 0.001, ****p < 0.0001; ns, not significant.

To determine the effect of farrerol on HCT116 cells, the cell viability of HCT116 cells treated with varying concentrations of farrerol was assessed using the CCK-8 assay. The results demonstrated that farrerol inhibited the proliferation of HCT116 cells in a dose-dependent manner. The half-maximal inhibitory concentration (IC50) of farrerol against HCT116 cells at 24 h was determined to be 72.11 μmol/L (Figure 7A). Based on these findings, concentrations of 40 μmol/L (low dose; approximately 0.56 times the 24-h IC50 value, sub-inhibitory concentration) and 80 μmol/L (high dose; slightly higher than the 24-h IC50 value, effective inhibitory concentration) were selected for subsequent treatments.

### 3.8 Farrerol inhibits the migration of CRC cells

To investigate the effect of farrerol on the migration of HCT116 cells, a wound healing assay was performed to assess the migratory ability of HCT116 cells treated with different concentrations of farrerol over 12 h and 24 h (Figures 7B,C). The results demonstrated that farrerol inhibited the migration of HCT116 cells in a dose-dependent and time-dependent manner.

### 3.9 Farrerol induces G0/G1 cell cycle arrest in CRC cells

**FIGURE 8:**
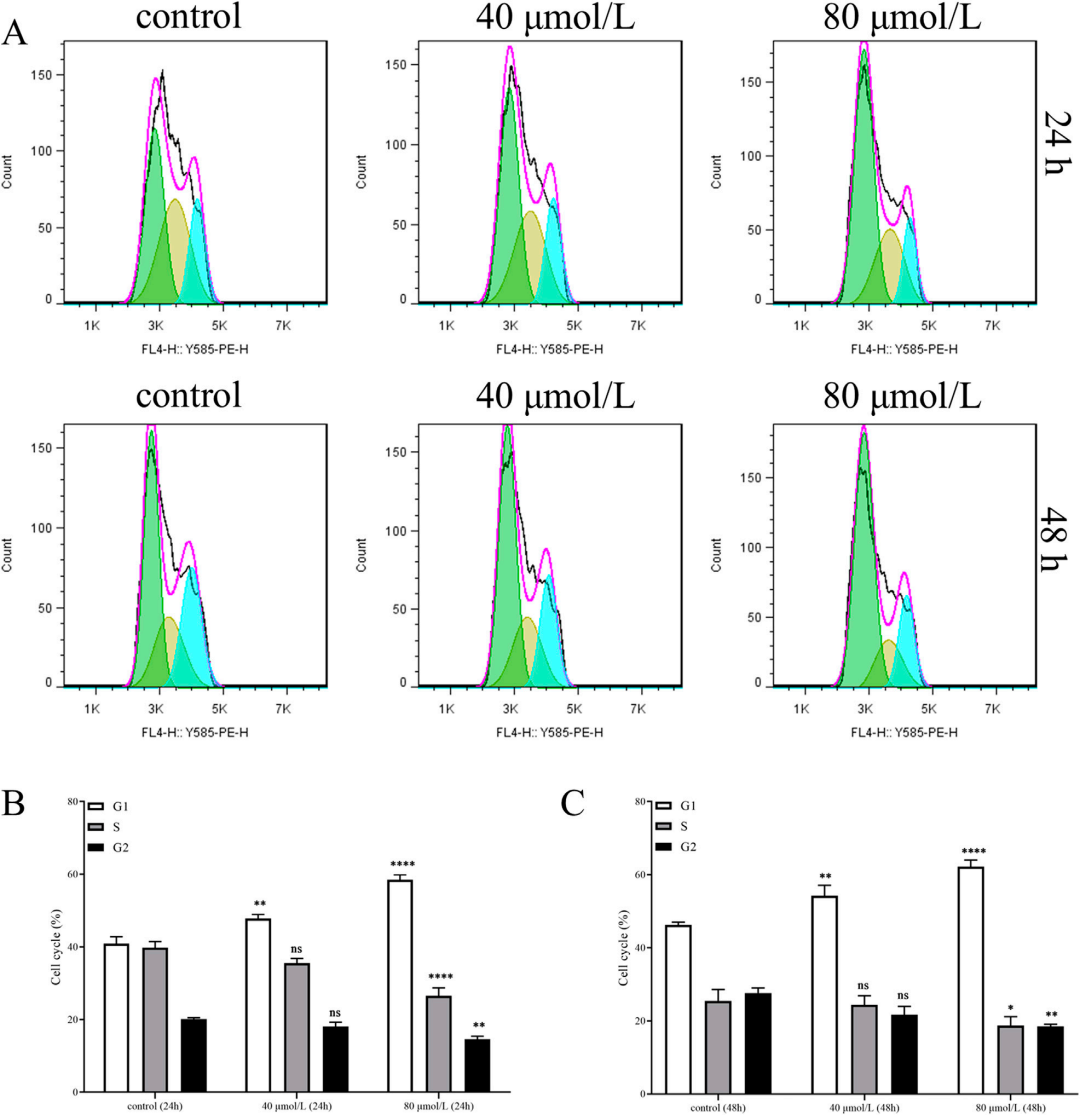
Farrerol induces cell cycle arrest in CRC Cells. **(A)** The cell cycle distribution of HCT116 cells, treated with different concentrations of farrerol for 24 and 48 h, was detected by flow cytometric analysis using propidium iodide staining. **(B,C)** Histograms showing the cell cycle distribution of HCT116 cells after 24 and 48 h of farrerol treatment. Data are presented as the mean ± standard deviation from three independent experiments. *p < 0.05, **p < 0.01, ***p < 0.001, ****p < 0.0001 vs. control group; ns, not significant.

To elucidate the mechanism by which farrerol inhibits cell proliferation, we analyzed the cell cycle distribution of CRC cells treated with farrerol using flow cytometry. The results demonstrated that farrerol significantly induced G0/G1 cell cycle arrest in HCT116 cells (Figures 8A–C). Furthermore, this cell cycle arrest became more pronounced with increasing concentrations of farrerol and longer treatment durations.

### 3.10 Farrerol affects the proliferation and migration of CRC cells via the VEGF signaling pathway

**FIGURE 9:**
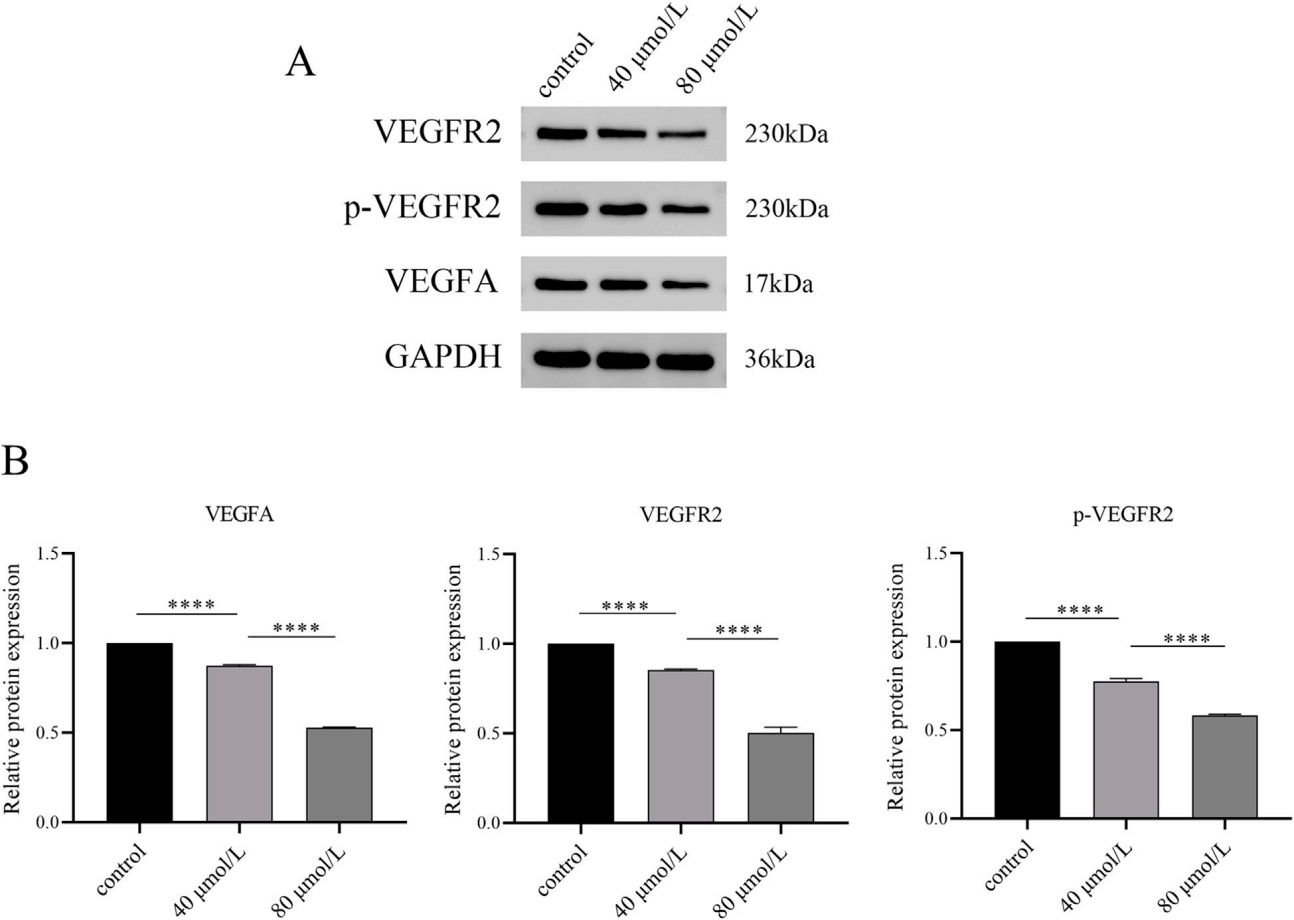
Western blot analysis of VEGF signaling pathway-related protein expression. **(A)** After HCT116 cells were treated with varying concentrations of farrerol for 24 h, the expression levels of GAPDH, VEGFA, VEGFR2, and p-VEGFR2 were detected by Western blot. **(B)** Semi-quantitative analysis was performed using GAPDH as the internal reference. *p < 0.05, **p < 0.01, ***p < 0.001, ****p < 0.0001; ns, not significant.

Based on network pharmacology and molecular dynamics simulations, we identified that farrerol potentially targets VEGFA and VEGFR2, with calculated binding free energies of −14.77 kcal·mol^-1^ and −22.78 kcal·mol^-1^, respectively. Therefore, we investigated whether this pathway is involved in the mechanism of action of farrerol against CRC. Western blot analysis revealed that farrerol reduced the protein levels of VEGFA and VEGFR2, accompanied by a corresponding decrease in phosphorylated VEGFR2 (p-VEGFR2). This inhibitory effect was more pronounced with increasing concentrations of farrerol (Figures 9A,B). These results indicate that farrerol may inhibit HCT-116 cell proliferation by binding to VEGFA and VEGFR2.

## 4 Discussion

CRC is a prevalent malignant neoplasm, and as the global incidence of this disease has gradually increased in recent years, the recurrence rate of tumors after surgery has also increased (Mejri et al., 2017; Patel et al., 2022). Although chemotherapy is the treatment of choice for most patients after surgery to prevent tumor recurrence, it can cause serious adverse effects and drug resistance while killing tumor cells. Consequently, researchers have endeavored to develop novel pharmaceuticals characterized by minimal adverse effects and drug resistance. Research has demonstrated that TCM has distinctive benefits in the realm of cancer treatment, characterized by its high efficiency and minimal adverse effects (Liu et al., 2020). Farrerol, an active ingredient of the TCM ' Rhododendron mariesii Hemsl. & E.H. Wilson’, has been demonstrated to possess potent anti-tumor properties in the treatment of gastric cancer, laryngeal squamous cell carcinoma and laryngeal squamous cell carcinoma (Li et al., 2019; Liu et al., 2016; Zhang et al., 2021). However, its potential in the treatment of CRC remains to be elucidated through further research. The mechanism of action of TCM in treating diseases has been demonstrated to involve multiple targets and multiple pathways. Consequently, the application of big data is needed to explore the targets and pathways of farrerol in treating CRC. The objective of this study was to ascertain the potential molecular mechanism of farrerol in treating CRC. This goal can be achieved by using network pharmacology in combination with molecular docking, thereby providing a theoretical foundation for the clinical application of farrerol and the study of CRC.

The KEGG enrichment results demonstrated that a multitude of disease pathways unrelated to the present study were enriched, in addition to those associated with CRC. This phenomenon may be attributable to the existence of shared molecular targets in the developmental process of disparate diseases. Consequently, the signaling pathways associated with CRC were selected for analysis. The PI3K-Akt signalling pathway, the VEGF signaling pathway, and the cell cycle may represent potential key mechanisms by which farrerol exerts its effects in the treatment of CRC. The PI3K/AKT signaling pathway represents a highly conserved signal transduction network in eukaryotic cells that promotes cell survival, cell growth and cell cycle progression (Glaviano et al., 2023). Aberrant expression or mutation of many components of the PI3K/AKT signaling pathway is associated with tumorigenesis in humans, and it is the most commonly activated pathway in human cancers (Yu et al., 2022; Yuan et al., 2023). The development and progression of cancer may be affected by a number of factors, including hyperactivation of membrane receptors (RTKs or GPCRs), mutation and amplification of PI3K (Triscott and Rubin, 2018; Voutsadakis, 2023), inactivation or deletion of the tumor suppressor gene PTEN (Bergholz et al., 2023; Chow and Baker, 2006), and/or overactivation of AKT (Altomare and Testa, 2005; Zhang et al., 2017). The KEGG results indicate that farrerol may act on RTK-related genes which may be one of its targets for the treatment of CRC.

Angiogenesis is a complex and dynamic process that is regulated by a variety of pro-angiogenic and anti-angiogenic molecules. These molecules play crucial roles in tumor growth, invasion and metastasis. VEGF plays an important role in vasculogenesis and angiogenesis (Ferrara and Adamis, 2016). In many human cancers, the development of tumor tissues is accompanied by the gradual formation of microenvironments, including conditions of hypoxia, ischemia and acidosis. These microenvironments release substantial quantities of growth factors and cytokines, which in turn stimulate the processes of angiogenesis and lymphangiogenesis. These processes are essential for the sustained growth and metabolic demands of the tumor (Gasparini et al., 2005). Bevacizumab, a humanized monoclonal antibody against VEGF, has been utilized in combination with chemotherapy as a first-line treatment for metastatic CRC, thereby demonstrating significant benefits for patients (Ferrara et al., 2004). In the course of KEGG enrichment analysis, we found that farrerol may affect VEGFA and KDR.

Abnormalities in cell cycle progression represent a fundamental mechanism of tumorigenesis, thus regulators of cell cycle machinery are rational targets for anti-cancer therapy. The cell cycle is comprises of four principal phases: Gap 1 (G1), DNA synthesis (S), Gap 2 (G2), and mitosis (M). The integrity and fidelity of DNA replication, and the precise and timely separation of sister chromatids, are subject to quality control mechanisms that are in place during the cell cycle. These include the G1/S checkpoint, which can be relieved through the sequential phosphorylation of Rb by Cyclin D-CDK4/6 and Cyclin E-CDK2, thereby facilitating the dissociation of E2F (Bertoli et al., 2013; Guardavaccaro and Pagano, 2006). The G2/M checkpoint is where the activity of the cyclin B-CDK1 kinase is subject to precise regulation by the Aurora A/PLK1/CDC25 and Chk1/WEE1 signaling pathways (O'Connell et al., 2000). The spindle assembly checkpoint is where the mitotic checkpoint complex inhibits the activity of the APC^Cdc20^ E3 ligase, thereby preventing the degradation of Cyclin B and Securin, and consequently halting mitotic progression (Lara-Gonzalez et al., 2012). According to the KEGG results, CCNA2, CCNA1, WEE1, CCNE1, CDK4, CHEK1, CDK2, AURKB, and CDC25B were enriched, suggesting that farrerol can act at both the G1/S checkpoint and the G2/M checkpoint.

The targets of farrerol and CRC were taken to intersected, resulting in the identification of 103 potential targets of farrerol for the treatment of CRC. Further screening of intersecting genes identified 12 core targets (CCNA1, CCNA2, CCNE1, CDK2, CDC25B, CYP19A1, HSP90AA1, ESR1, ESR2, RAF1, SRC, and PTPN1). An analysis of the KEGG results revealed that farrerol is capable of acting on VEGFA and KDR targets, which have been established as key targets of the VEGF signaling pathway. Consequently, the decision was made to incorporate these two targets in the subsequent study. Elevated expression of CCNA1 has been strongly associated with resistance to paclitaxel, doxorubicin and 5-fluorouracil in human ovarian cancer cells (Huang et al., 2016). CCNA2 was found to promote cell proliferation in cells from early-stage CRC; however, in later stage CRC, it was expressed at lower levels but promoted cell invasiveness (Guo et al., 2021). CCNE1 has been shown to form a complex with CDK2 and to function as a regulatory subunit of CDK2, whose activity is required for the cell cycle G1/S transition. Overactivation of CDK2 has been demonstrated to lead to excessive phosphorylation of Rb, resulting in an unregulated and premature G1-S transition (Tadesse et al., 2020). CDC25B is a dual specificity phosphatase that functions in the removal of certain inhibitory phosphate groups. These groups are required for the assembly of the CDK1-cyclin B (CCNB) complex (Timofeev et al., 2010).

CYP19A1, a cytochrome P450 monooxygenase, is located in the endoplasmic reticulum and catalyzes the conversion of androgens to estrogens, thereby participating in the final step of sex hormone biosynthesis (Baravalle et al., 2017). A preceding study demonstrated that approximately 20% of patients diagnosed with gastric cancer exhibited an excess of the CYP19A1 gene (Kobayashi et al., 2019). Silencing of the CYP19A1 gene has been demonstrated to exert a growth-inhibiting effect on gastric cancer cells (Ma et al., 2022). Inhibition of CYP19A1 resulted in the downregulation of PD-L1, IL-6 and TGF-β expression in colon cancer cells, thereby enhancing the tumor-killing ability of CD8^+^ T cells (Liu et al., 2023). HSP90AA1 is an inducible molecular chaperone that promotes maturation, structural maintenance, and appropriate regulation of specific target proteins. Studies have demonstrated that elevated levels of HSP90 AA1 expression in CRC tissues are associated with a poorer patient prognosis and may be considered a potential independent prognostic factor for CRC patients (Zhang et al., 2019).

ESR1 and ESR2 are both classified as members of the nuclear hormone receptor family. Following binding with estrogen, the activation of intracellular signal transduction cascades is observed. In metastatic breast cancer, ESR1 mutations are frequently acquired during aromatase inhibitor therapy and may play a role in metastatic progression (Dustin et al., 2019). In the colonic mucosa, estrogen can selectively activate ESR2-mediated regulation of mismatch repair proteins, and pro-apoptotic signaling, and can modulate the inflammatory tumor microenvironment and various immune surveillance mechanisms, thereby exerting anti-tumor effects. Consequently, the loss of ESR2 in colorectal tissue may promote tumor development (Caiazza et al., 2015). RAF1 belongs to the serine/threonine kinase family and is part of the Ras-MAPK signal transduction cascade. The Ras-MAPK cascade is associated with proliferation, differentiation, apoptosis, survival, and carcinogenic transformation. A study showed that low expression of RAF1, or the use of RAF1 inhibitors, significantly reduces the clonogenic and tumorigenic capacity of CRC cells (Borovski et al., 2017).

The Src proto-oncogene is a protein tyrosine kinase that plays a critical role in various cellular processes, including cell growth, division, migration, and survival signaling pathways. In advanced CRC, defects in kinase regulation and substrate degradation have been shown to result in high oncogenic potential in wild-type Src expression, promoting tumor growth and liver metastasis in nude mice (Leroy et al., 2009). The suppression of Src expression has shown efficacy in the inhibition of the growth, migration and invasion of 5-FU-resistant colon cancer cells (Ahn et al., 2015). PTPN1 belongs to the class of protein tyrosine phosphatases, enzymes that catalyze the removal of phosphate groups from tyrosine residues. In CRC tissues, PTPN1 expression is significantly elevated, and patients with higher PTPN1 expression have lower survival rates (Chen et al., 2014).

The molecular docking results revealed that farrerol could spontaneously bind to the core targets. Except for RAF1 and ESR1, the binding energies of the remaining targets were less than 5, indicating that farrerol could bind to these targets with strong activity and stable structure. These findings indicate that farrerol is capable of modulating the biological activity of CRC-related targets. The molecular dynamics simulations revealed that both the KDR-farrerol and VEGFA-farrerol complex systems exhibited stability, with favorable hydrogen bonding interactions present within the complexes.

By integrating network pharmacology, molecular docking, and molecular dynamics simulation analyses, we identified VEGFA and VEGFR2 as key targets of farrerol in the treatment of CRC, which were subsequently validated through *in vitro* experiments. To the best of our knowledge, this study is the first to report the inhibitory effect of farrerol on CRC cells *in vitro*. Cell cycle arrest is a major mechanism for inhibiting cell growth. Previous studies have shown that farrerol suppresses the growth of various cancers by inducing cell cycle arrest (Guo et al., 2022; Liu et al., 2016). Consistent with these findings, the present study demonstrated that farrerol induced G0/G1 phase cell cycle arrest in HCT116 cells. Notably, a mild G1 arrest was observed in the low-dose group at 24 h, which became markedly enhanced at 48 h; in contrast, a significant arrest was already evident in the high-dose group at 24 h and was maintained at a similar intensity at 48 h, suggesting that once established, the arrest persists. However, the underlying mechanisms require further investigation.

VEGF belongs to a family of proteins that include VEGF-A, VEGF-B, VEGF-C, VEGF-D, VEGF-E (encoded by viruses) and placental growth factor (PlGF). Among these factors, VEGF-A plays a dominant role in tumor angiogenesis, mainly through KDR. The Serum and plasma levels of VEGFA in CRC patients have been shown to be approximately twice as high as those in healthy patients (Kut et al., 2007). In a phase 3 clinical trial, the median overall survival (mOS) for patients with metastatic colorectal cancer (mCRC) treated with the IFL regimen (bolus 5-FU, leucovorin, and irinotecan) was 15.6 months. However, for patients receiving IFL + bevacizumab as first-line therapy, the mOS increased to 20.3 months, indicating that VEGF inhibitors can improve patient survival (Hurwitz et al., 2004). In CRC tissue, the expression level of KDR is significantly greater than that adjacent normal tissue. The inhibition of KDR with emodin results in the growth of CRC cells (Dai et al., 2019). This study identified farrerol as a novel inhibitor of the VEGF signaling pathway. Farrerol was found to reduce the levels of VEGFA and VEGFR2. Following VEGFR2 inhibition, its phosphorylation level was correspondingly decreased, indicating suppression of VEGF signaling pathway activation.

We found that farrerol can induce cell cycle arrest, which may have a plausible mechanistic association with the VEGF signaling pathway. Key downstream effectors of the VEGF signaling pathway, such as the PI3K/AKT and RAS/RAF/ERK pathways, serve as central hubs regulating the cell cycle—particularly the G1/S transition and G2/M progression. Therefore, inhibition of upstream VEGFR2 by farrerol leads to downregulation of these pro-survival/proliferation signals, thereby indirectly inducing cell cycle arrest. In addition, molecular docking results indicated that farrerol exhibits favorable binding affinity with cyclins such as CCNA1, CCNA2, CCNE1, and CDK2, which may also partially account for farrerol-induced cell cycle arrest. Further studies are warranted to validate these findings.

Placing the findings of this study within the broader context of natural polyphenols in anti-CRC research is of significant importance. Numerous natural polyphenols, such as luteolin and quercetin, have been extensively reported to inhibit the proliferation of colorectal cancer cells through mechanisms including cell cycle regulation and apoptosis induction (Yang et al., 2016; Yoo et al., 2022). Notably, this study found that farrerol induced significant G0/G1 phase cell cycle arrest in HCT-116 cells, which is consistent with the G0/G1 phase arrest reported for luteolin in the same cell line, suggesting a similar mechanism of action (Jang et al., 2019). In terms of potency comparison, the 24-h IC50 value of farrerol against HCT-116 cells was determined to be 72.11 μmol/L in this study. This value falls within the typical activity range of natural polyphenolic compounds but is higher than that of more potent flavonoids such as quercetin (24-h IC50 value of 54.04 μmol/L) (Fosso et al., 2023). These comparative results not only demonstrate the clear potential of farrerol as a natural flavonoid in anti-CRC activity but also suggest that its bioactivity may be further enhanced through structural optimization. In summary, compared with other natural polyphenols, farrerol exhibits a unique profile of action, providing a theoretical basis for further investigation into its role as a candidate drug for CRC treatment.

In summary, this study predicted farrerol as a potential inhibitor of the VEGF signaling pathway through computational screening, and subsequently observed its inhibition of HCT-116 cell proliferation along with downregulation of VEGF signaling pathway activity in a series of experiments. Although the findings are encouraging, several limitations of this study should be considered. In the wound healing assay, although experiments were conducted under serum-free conditions to inhibit cell proliferation, specific anti-proliferative agents such as mitomycin C were not employed. Therefore, the observed wound closure effect reflects a combination of cell migration and residual proliferative activity, rather than pure cell migration behavior. This methodological limitation should be taken into account when interpreting the results. Furthermore, it should be noted that although these findings form a continuous chain of evidence, they remain primarily at the level of “correlation”. The mechanism linking computational predictions to cellular phenotypes is complex. For instance, the proliferation inhibition and weakened downstream signaling induced by farrerol may result from direct inhibition of VEGFR, or may be indirectly achieved through effects on other upstream receptors or parallel pathways. Therefore, fully attributing the observed phenotype to direct inhibition of VEGFR remains a hypothesis requiring further validation at this stage. Future research will be crucial, including validation of functional dependency in more stringent genetic models (such as VEGFR knock down or knock out), determination of VEGFA and VEGFR mRNA levels, apoptosis assays, and rescue experiments using specific pathway agonists, to ultimately establish the causal relationship regarding farrerol’s target and mechanism. Moreover, this study was limited to the HCT116 cell line; until validation is completed in other CRC models or *in vivo* systems, these findings should be regarded as preliminary. The significance of this study lies in providing a clear and testable mechanistic hypothesis, along with a solid preliminary experimental foundation, for a promising natural product.

## 5 Conclusion

This study, for the first time, validated the antitumor effect of farrerol against CRC through network pharmacology, molecular docking, molecular dynamics simulations, and *in vitro* experiments. Network pharmacology, molecular docking, and molecular dynamics simulations theoretically supported the potential of farrerol in treating CRC at the computational level. The results indicated that farrerol may exert its antitumor effects by modulating the VEGF signaling pathway and the cell cycle. *In vitro* experiments demonstrated that farrerol regulates the VEGF signaling pathway through binding to VEGFA and its receptor VEGFR2. Additionally, farrerol was found to induce G0/G1 phase cell cycle arrest.

## Data Availability

The original contributions presented in the study are included in the article/Supplementary Material, further inquiries can be directed to the corresponding author.
