# Supplementary figures and images for: Farrerol inhibits proliferation and migration of colorectal cancer via the VEGF signaling pathway: evidence from network pharmacology, molecular docking, molecular dynamics simulation, and in vitro experiments
Source: Front Pharmacol. 2025 Dec 3;16:1717293. doi: 10.3389/fphar.2025.1717293 (PMC12708557; doi:10.3389/fphar.2025.1717293)

| Protein Name \ Number of repetitions | Number of repetitions                                                               |                                                                                      |                                                                                       |
|--------------------------------------|-------------------------------------------------------------------------------------|--------------------------------------------------------------------------------------|---------------------------------------------------------------------------------------|
|                                      | 1                                                                                   | 2                                                                                    | 3                                                                                     |
| GAPDH                                | 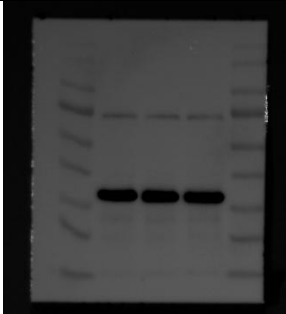   | 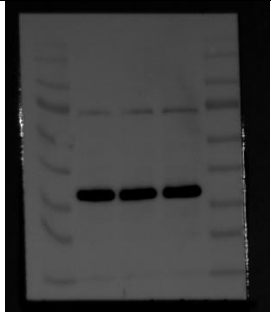   | 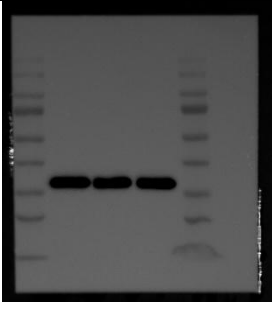   |
| VEGFA                                | 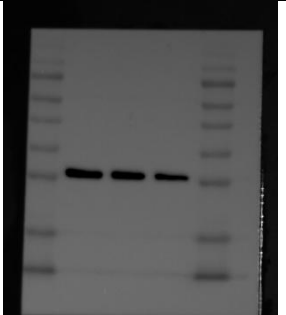  | 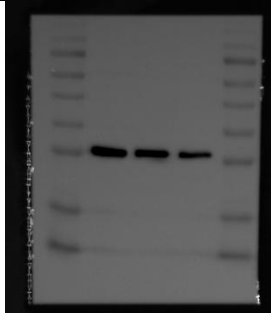  | 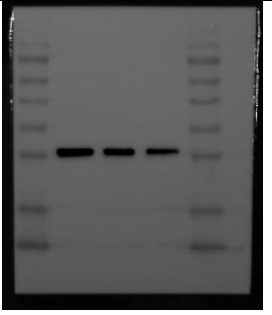  |
| VEGFR2                               | 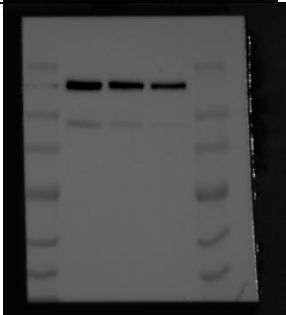 | 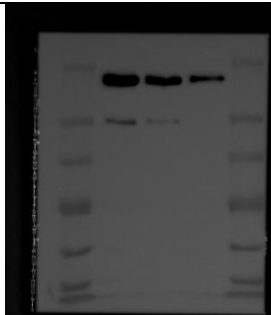 | 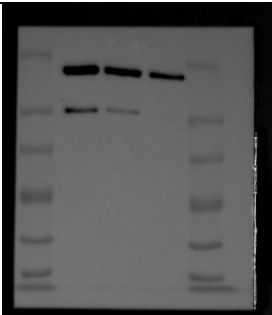 |
| p-VEGFR2                             | 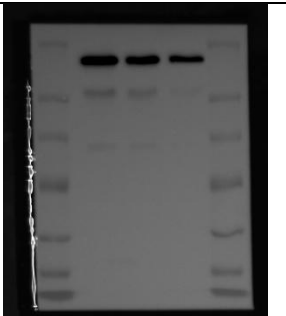 | 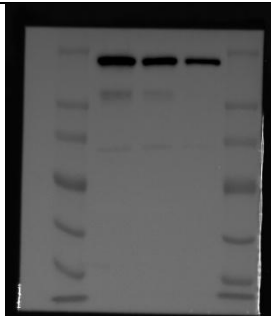 | 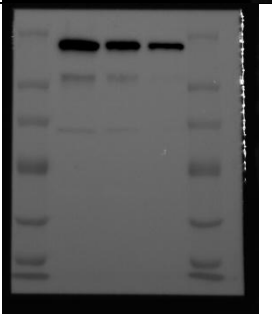 |

Supplement: Supplementary file 1 [file DataSheet2.pdf]
