# Supplementary material for: Farrerol inhibits proliferation and migration of colorectal cancer via the VEGF signaling pathway: evidence from network pharmacology, molecular docking, molecular dynamics simulation, and in vitro experiments
Source: Front Pharmacol. 2025 Dec 3;16:1717293. doi: 10.3389/fphar.2025.1717293 (PMC12708557; doi:10.3389/fphar.2025.1717293)
Supplement: Supplementary file 2 [file DataSheet1.pdf]

| Category | Term | Count | Gene Ratio | PValue | Genes | List Total | Pop Hits | Pop Total | Fold Enrichment | Bonferroni | Benjamini | FDR |
|---|---|---|---|---|---|---|---|---|---|---|---|---|
| KEGG_PATHWAY | hsa04071:Sphingolipid signaling pathway | 14 | 0.13592233 | 6.05288E-10 | ABCC1, ROCK1, PRKCB, NOS3, SPHK2, PDPK1, SPHK1, PRKCE, TNF, MAPK11, ADORA3, ADORA1, R | 98 | 122 | 8840 | 10.35128806 | 1.43453E-07 | 8.96524E-08 | 5.74986E-08 |
| KEGG_PATHWAY | hsa04370:VEGF signaling pathway | 11 | 0.106796117 | 7.5656E-10 | MAPK11, SPHK2, SRC, PRKCB, NOS3, SPHK1, MAPKAPK2, KDR, RAF1, PTGS2, VEGFA | 98 | 60 | 8840 | 16.53741497 | 1.79305E-07 | 8.96524E-08 | 5.74986E-08 |
| KEGG_PATHWAY | hsa05200:Pathways in cancer | 24 | 0.233009709 | 9.299E-09 | HSP90AA1, ROCK1, PRKCB, PTGER3, BRAF, PTGS2, ESR1, PGF, ESR2, IGF1R, VEGFA, CCNA2, CCNA1 | 98 | 533 | 8840 | 4.06172225 | 2.20386E-06 | 7.34621E-07 | 4.71149E-07 |
| KEGG_PATHWAY | hsa00910:Nitrogen metabolism | 7 | 0.067961165 | 1.67656E-08 | CA12, CA1, CA2, CA4, CA7, CA6, CA9 | 98 | 17 | 8840 | 37.14285714 | 3.97344E-06 | 9.93363E-07 | 6.37094E-07 |
| KEGG_PATHWAY | hsa05205:Proteoglycans in cancer | 15 | 0.145631068 | 4.03261E-08 | ROCK1, PRKCB, PDPK1, SRC, BRAF, ESR1, TNF, IGF1R, VEGFA, MAPK11, RPS6KB1, CTSL, KDR, RAF1, | 98 | 204 | 8840 | 6.632653061 | 9.55724E-06 | 1.91146E-06 | 1.22591E-06 |
| KEGG_PATHWAY | hsa04914:Progesterone-mediated oocyte maturation | 11 | 0.106796117 | 3.26603E-07 | CCNA2, MAPK11, CCNA1, HSP90AA1, CDK2, BRAF, PGR, RAF1, CDC25B, AURKA, IGF1R | 98 | 111 | 8840 | 8.939143225 | 7.74018E-05 | 1.29008E-05 | 8.27393E-06 |
| KEGG_PATHWAY | hsa04913:Ovarian steroidogenesis | 8 | 0.077669903 | 1.20152E-06 | INSR, HSD17B1, HSD17B2, AKR1C3, CYP1B1, PTGS2, CYP19A1, IGF1R | 98 | 51 | 8840 | 14.14965986 | 0.000284719 | 4.068E-05 | 2.60901E-05 |
| KEGG_PATHWAY | hsa04933:AGE-RAGE signaling pathway in diabetic complications | 10 | 0.097087379 | 1.43971E-06 | MAPK11, PRKCB, CDK4, NOS3, PRKCE, SERPINE1, NOX4, TNF, F3, VEGFA | 98 | 101 | 8840 | 8.931097191 | 0.000341154 | 4.26515E-05 | 2.73546E-05 |
| KEGG_PATHWAY | hsa01521:EGFR tyrosine kinase inhibitor resistance | 9 | 0.087378641 | 2.3781E-06 | RPS6KB1, SRC, PRKCB, KDR, BRAF, RAF1, MET, IGF1R, VEGFA | 98 | 80 | 8840 | 10.14795918 | 0.000563451 | 6.26233E-05 | 4.01634E-05 |
| KEGG_PATHWAY | hsa04015:Rap1 signaling pathway | 13 | 0.126213592 | 3.1483E-06 | PRKCB, SRC, INSR, BRAF, PGF, IGF1R, VEGFA, MAPK11, CNR1, KDR, RAF1, DRD2, MET | 98 | 212 | 8840 | 5.531382364 | 0.000745869 | 7.46146E-05 | 4.78541E-05 |
| KEGG_PATHWAY | hsa04151:PI3K-Akt signaling pathway | 16 | 0.155339806 | 8.25819E-06 | HSP90AA1, NOS3, PDPK1, INSR, PGF, IGF1R, VEGFA, RXRA, RPS6KB1, CCNE1, CDK4, CDK2, KDR, R/ | 98 | 362 | 8840 | 3.986920735 | 0.001955284 | 0.000177926 | 0.000114113 |
| KEGG_PATHWAY | hsa01522:Endocrine resistance | 9 | 0.087378641 | 1.18413E-05 | MAPK11, RPS6KB1, SRC, CDK4, BRAF, RAF1, ESR1, ESR2, IGF1R | 98 | 99 | 8840 | 8.200371058 | 0.00280247 | 0.000233866 | 0.00014999 |
| KEGG_PATHWAY | hsa04210:Apoptosis | 10 | 0.097087379 | 1.68329E-05 | ERN1, TUBA1A, PARP1, PDPK1, CTSL, CTSK, PRF1, RAF1, TNF, CTSD | 98 | 136 | 8840 | 6.632653061 | 0.003981495 | 0.000306878 | 0.000196816 |
| KEGG_PATHWAY | hsa05207:Chemical carcinogenesis - receptor activation | 12 | 0.116504854 | 2.16695E-05 | AR, HSP90AA1, RXRA, RPS6KB1, SRC, PRKCB, CYP1B1, PGR, RAF1, ESR1, ESR2, VEGFA | 98 | 215 | 8840 | 5.034646417 | 0.005122552 | 0.000366833 | 0.000235268 |
| KEGG_PATHWAY | hsa04218:Cellular senescence | 10 | 0.097087379 | 5.26317E-05 | CCNA2, MAPK11, CCNA1, CCNE1, CDK4, CHEK1, CDK2, MAPKAPK2, SERPINE1, RAF1 | 98 | 157 | 8840 | 5.745482907 | 0.012396574 | 0.000831582 | 0.000533335 |
| KEGG_PATHWAY | hsa05161:Hepatitis B | 10 | 0.097087379 | 7.04969E-05 | CCNA2, MAPK11, CCNA1, CCNE1, SRC, PRKCB, CDK2, BRAF, RAF1, TNF | 98 | 163 | 8840 | 5.533992738 | 0.016569548 | 0.00100711 | 0.00064591 |
| KEGG_PATHWAY | hsa04510:Focal adhesion | 11 | 0.106796117 | 7.22399E-05 | ROCK1, PDPK1, SRC, PRKCB, KDR, BRAF, RAF1, MET, PGF, IGF1R, VEGFA | 98 | 203 | 8840 | 4.887905901 | 0.016975743 | 0.00100711 | 0.00064591 |
| KEGG_PATHWAY | hsa05215:Prostate cancer | 8 | 0.077669903 | 9.43893E-05 | AR, HSP90AA1, PDPK1, CCNE1, CDK2, BRAF, RAF1, IGF1R | 98 | 98 | 8840 | 7.363598501 | 0.022122947 | 0.001242793 | 0.000797066 |
| KEGG_PATHWAY | hsa04010:MAPK signaling pathway | 13 | 0.126213592 | 0.00010087 | PRKCB, INSR, BRAF, TNF, PGF, CDC25B, IGF1R, VEGFA, MAPK11, MAPKAPK2, KDR, RAF1, MET | 98 | 300 | 8840 | 3.908843537 | 0.023623959 | 0.001258225 | 0.000806963 |
| KEGG_PATHWAY | hsa05223:Non-small cell lung cancer | 7 | 0.067961165 | 0.000140599 | RXRA, PDPK1, PRKCB, CDK4, BRAF, RAF1, MET | 98 | 73 | 8840 | 8.649706458 | 0.032775077 | 0.001666093 | 0.001068549 |
| KEGG_PATHWAY | hsa05163:Human cytomegalovirus infection | 11 | 0.106796117 | 0.000175734 | MAPK11, RPS6KB1, ROCK1, SRC, PRKCB, CDK4, PTGER3, RAF1, PTGS2, TNF, VEGFA | 98 | 226 | 8840 | 4.39046415 | 0.040797109 | 0.0018202 | 0.001167386 |
| KEGG_PATHWAY | hsa05208:Chemical carcinogenesis - reactive oxygen species | 11 | 0.106796117 | 0.000175734 | MAPK11, PTPN1, PDPK1, SRC, AKR1C3, CYP1B1, NOX4, BRAF, RAF1, MET, VEGFA | 98 | 226 | 8840 | 4.39046415 | 0.040797109 | 0.0018202 | 0.001167386 |
| KEGG_PATHWAY | hsa04931:Insulin resistance | 8 | 0.077669903 | 0.000184324 | PTPN1, RPS6KB1, PDPK1, PRKCB, NOS3, INSR, PRKCE, TNF | 98 | 109 | 8840 | 6.620483056 | 0.04274822 | 0.0018202 | 0.001167386 |
| KEGG_PATHWAY | hsa04066:HIF-1 signaling pathway | 8 | 0.077669903 | 0.000184324 | RPS6KB1, PRKCB, NOS3, INSR, SERPINE1, IGF1R, PDK1, VEGFA | 98 | 109 | 8840 | 6.620483056 | 0.04274822 | 0.0018202 | 0.001167386 |
| KEGG_PATHWAY | hsa04726:Serotonergic synapse | 8 | 0.077669903 | 0.000256939 | APP, MAOB, PRKCB, ALOX15, BRAF, RAF1, PTGS2, PTGS1 | 98 | 115 | 8840 | 6.275066548 | 0.059084813 | 0.002435778 | 0.001562187 |
| KEGG_PATHWAY | hsa04148:Efferocytosis | 9 | 0.087378641 | 0.000300649 | MAPK11, RXRA, SPHK2, SPHK1, ALOX15, MAPKAPK2, PPARG, SGK1, PTGS2 | 98 | 156 | 8840 | 5.204081633 | 0.068784498 | 0.002740532 | 0.001757641 |
| KEGG_PATHWAY | hsa04150:mTOR signaling pathway | 9 | 0.087378641 | 0.00032776 | RPS6KB1, PDPK1, PRKCB, INSR, BRAF, RAF1, SGK1, TNF, IGF1R | 98 | 158 | 8840 | 5.138207182 | 0.074750598 | 0.002774258 | 0.001779271 |
| KEGG_PATHWAY | hsa04110:Cell cycle | 9 | 0.087378641 | 0.00032776 | CCNA2, CCNA1, WEE1, CCNE1, CDK4, CHEK1, CDK2, AURKB, CDC25B | 98 | 158 | 8840 | 5.138207182 | 0.074750598 | 0.002774258 | 0.001779271 |
| KEGG_PATHWAY | hsa04152:AMPK signaling pathway | 8 | 0.077669903 | 0.000369097 | CCNA2, CCNA1, RPS6KB1, PDPK1, HNF4A, INSR, PPARG, IGF1R | 98 | 122 | 8840 | 5.915021746 | 0.083773983 | 0.003016416 | 0.001934579 |
| KEGG_PATHWAY | hsa00590:Arachidonic acid metabolism | 6 | 0.058252427 | 0.000524872 | ALOX15, PLA2G2A, PLA2G10, AKR1C3, PTGS2, PTGS1 | 98 | 61 | 8840 | 8.87253262 | 0.116997614 | 0.004146491 | 0.002659353 |
| KEGG_PATHWAY | hsa00140:Steroid hormone biosynthesis | 6 | 0.058252427 | 0.000566006 | HSD17B1, HSD17B2, AKR1C3, HSD17B3, CYP1B1, CYP19A1 | 98 | 62 | 8840 | 8.729427255 | 0.125568637 | 0.00432721 | 0.002775257 |
| KEGG_PATHWAY | hsa04068:FoxO signaling pathway | 8 | 0.077669903 | 0.000594145 | MAPK11, PDPK1, INSR, CDK2, BRAF, RAF1, SGK1, IGF1R | 98 | 132 | 8840 | 5.466914038 | 0.131384032 | 0.004400385 | 0.002822188 |
| KEGG_PATHWAY | hsa04270:Vascular smooth muscle contraction | 8 | 0.077669903 | 0.000650064 | EDNRA, ROCK1, PRKCB, PRKCE, PLA2G2A, PLA2G10, BRAF, RAF1 | 98 | 134 | 8840 | 5.385318306 | 0.142826732 | 0.00466864 | 0.002994233 |
| KEGG_PATHWAY | hsa04960:Aldosterone-regulated sodium reabsorption | 5 | 0.048543689 | 0.000755479 | PDPK1, PRKCB, INSR, SGK1, NR3C2 | 98 | 38 | 8840 | 11.86895811 | 0.163991161 | 0.00526613 | 0.003377434 |
| KEGG_PATHWAY | hsa04915:Estrogen signaling pathway | 8 | 0.077669903 | 0.000808284 | HSP90AA1, SRC, NOS3, PGR, RAF1, ESR1, CTSD, ESR2 | 98 | 139 | 8840 | 5.191601821 | 0.174396622 | 0.005473238 | 0.003510262 |
| KEGG_PATHWAY | hsa05418:Fluid shear stress and atherosclerosis | 8 | 0.077669903 | 0.00087951 | MAPK11, HSP90AA1, SRC, CTSL, NOS3, KDR, TNF, VEGFA | 98 | 141 | 8840 | 5.117962078 | 0.188227835 | 0.005790107 | 0.003713486 |
| KEGG_PATHWAY | hsa05219:Bladder cancer | 5 | 0.048543689 | 0.001010758 | SRC, CDK4, BRAF, RAF1, VEGFA | 98 | 41 | 8840 | 11.00049776 | 0.213113216 | 0.006474318 | 0.004152305 |
| KEGG_PATHWAY | hsa04014:Ras signaling pathway | 10 | 0.097087379 | 0.001163312 | PRKCB, INSR, PLA2G2A, PLA2G10, KDR, RAF1, MET, PGF, IGF1R, VEGFA | 98 | 238 | 8840 | 3.790087464 | 0.241084967 | 0.007102127 | 0.004554951 |
| KEGG_PATHWAY | hsa05224:Breast cancer | 8 | 0.077669903 | 0.001168704 | RPS6KB1, CDK4, BRAF, PGR, RAF1, ESR1, ESR2, IGF1R | 98 | 148 | 8840 | 4.875896304 | 0.242055367 | 0.007102127 | 0.004554951 |
| KEGG_PATHWAY | hsa05226:Gastric cancer | 8 | 0.077669903 | 0.001263717 | RXRA, RPS6KB1, TERT, CCNE1, CDK2, BRAF, RAF1, MET | 98 | 150 | 8840 | 4.810884354 | 0.25895237 | 0.007487524 | 0.004802125 |
| KEGG_PATHWAY | hsa05203:Viral carcinogenesis | 9 | 0.087378641 | 0.001802301 | CCNA2, POLB, CCNA1, CCNE1, SRC, CDK4, CHEK1, CDK2, MAPKAPK2 | 98 | 205 | 8840 | 3.960179194 | 0.347882635 | 0.010418179 | 0.006681701 |
| KEGG_PATHWAY | hsa05206:MicroRNAs in cancer | 11 | 0.106796117 | 0.002151935 | ABCC1, ROCK1, CCNE1, PRKCB, PRKCE, CYP1B1, RAF1, PTGS2, MET, CDC25B, VEGFA | 98 | 312 | 8840 | 3.180272109 | 0.399839551 | 0.012143064 | 0.007787957 |
| KEGG_PATHWAY | hsa04022:cGMP-PKG signaling pathway | 8 | 0.077669903 | 0.002260379 | EDNRA, ROCK1, NOS3, INSR, ADORA3, PRKCE, ADORA1, RAF1 | 98 | 166 | 8840 | 4.347184657 | 0.415101108 | 0.01245837 | 0.007990178 |
| KEGG_PATHWAY | hsa05417:Lipid and atherosclerosis | 9 | 0.087378641 | 0.002499441 | ERN1, MAPK11, HSP90AA1, RXRA, PDPK1, SRC, NOS3, PPARG, TNF | 98 | 216 | 8840 | 3.758503401 | 0.447393483 | 0.013462899 | 0.008634433 |
| KEGG_PATHWAY | hsa05225:Hepatocellular carcinoma | 8 | 0.077669903 | 0.002585268 | RPS6KB1, TERT, PRKCB, CDK4, BRAF, RAF1, MET, IGF1R | 98 | 170 | 8840 | 4.244897959 | 0.458548499 | 0.013615742 | 0.008732459 |
| KEGG_PATHWAY | hsa05166:Human T-cell leukemia virus 1 infection | 9 | 0.087378641 | 0.003043108 | CCNA2, POLB, CCNA1, TERT, CCNE1, CDK4, CHEK1, CDK2, TNF | 98 | 223 | 8840 | 3.640523474 | 0.514373826 | 0.01567862 | 0.010055487 |
| KEGG_PATHWAY | hsa04540:Gap junction | 6 | 0.058252427 | 0.00332259 | TUBB2B, TUBA1A, SRC, PRKCB, DRD2, RAF1 | 98 | 92 | 8840 | 5.882874889 | 0.545594332 | 0.016754337 | 0.010745398 |
| KEGG_PATHWAY | hsa05165:Human papillomavirus infection | 11 | 0.106796117 | 0.003447919 | CCNA2, CCNA1, RPS6KB1, TERT, CCNE1, CDK4, CDK2, RAF1, PTGS2, TNF, VEGFA | 98 | 333 | 8840 | 2.979714408 | 0.558937549 | 0.016838488 | 0.010799368 |
| KEGG_PATHWAY | hsa04520:Adherens junction | 6 | 0.058252427 | 0.003481375 | PTPN1, ROCK1, SRC, INSR, MET, IGF1R | 98 | 93 | 8840 | 5.81961817 | 0.56243302 | 0.016838488 | 0.010799368 |
| KEGG_PATHWAY | hsa04923:Regulation of lipolysis in adipocytes | 5 | 0.048543689 | 0.003908741 | INSR, PTGER3, ADORA1, PTGS2, PTGS1 | 98 | 59 | 8840 | 7.644413698 | 0.604730265 | 0.018527433 | 0.011882573 |
| KEGG_PATHWAY | hsa04114:Oocyte meiosis | 7 | 0.067961165 | 0.004156558 | MAPK11, AR, CCNE1, CDK2, PGR, AURKA, IGF1R | 98 | 139 | 8840 | 4.542651593 | 0.627365481 | 0.019315768 | 0.012388172 |
| KEGG_PATHWAY | hsa04371:Apelin signaling pathway | 7 | 0.067961165 | 0.004304912 | RPS6KB1, SPHK2, NOS3, SPHK1, PRKCE, SERPINE1, RAF1 | 98 | 140 | 8840 | 4.510204082 | 0.64029342 | 0.019488564 | 0.012498995 |
| KEGG_PATHWAY | hsa04666:Fc gamma R-mediated phagocytosis | 6 | 0.058252427 | 0.004358202 | RPS6KB1, SPHK2, PRKCB, SPHK1, PRKCE, RAF1 | 98 | 98 | 8840 | 5.522698875 | 0.644827346 | 0.019488564 | 0.012498995 |
| KEGG_PATHWAY | hsa04625:C-type lectin receptor signaling pathway | 6 | 0.058252427 | 0.005836259 | MAPK11, SRC, MAPKAPK2, RAF1, PTGS2, TNF | 98 | 105 | 8840 | 5.15451895 | 0.750237682 | 0.025614693 | 0.016427989 |
| KEGG_PATHWAY | hsa05221:Acute myeloid leukemia | 5 | 0.048543689 | 0.006489925 | CCNA2, CCNA1, RPS6KB1, BRAF, RAF1 | 98 | 68 | 8840 | 6.632653061 | 0.786287787 | 0.027965675 | 0.017935792 |
| KEGG_PATHWAY | hsa04020:Calcium signaling pathway | 9 | 0.087378641 | 0.006642057 | EDNRA, SPHK2, PRKCB, NOS3, SPHK1, PTGER3, KDR, MET, VEGFA | 98 | 254 | 8840 | 3.196207617 | 0.79390513 | 0.028110135 | 0.018028441 |
| KEGG_PATHWAY | hsa04917:Prolactin signaling pathway | 5 | 0.048543689 | 0.007552693 | MAPK11, SRC, RAF1, ESR1, ESR2 | 98 | 71 | 8840 | 6.352400115 | 0.834168357 | 0.031403305 | 0.020140516 |
| KEGG_PATHWAY | hsa05218:Melanoma | 5 | 0.048543689 | 0.008321422 | CDK4, BRAF, RAF1, MET, IGF1R | 98 | 73 | 8840 | 6.178361756 | 0.861989918 | 0.034003053 | 0.021807865 |
| KEGG_PATHWAY | hsa04928:Parathyroid hormone synthesis, secretion and action | 6 | 0.058252427 | 0.008516344 | RXRA, MMP13, PRKCB, BRAF, RAF1, SGK1 | 98 | 115 | 8840 | 4.706299911 | 0.86827214 | 0.03420972 | 0.021940411 |
| KEGG_PATHWAY | hsa04115:p53 signaling pathway | 5 | 0.048543689 | 0.00913981 | CCNE1, CDK4, CHEK1, CDK2, SERPINE1 | 98 | 75 | 8840 | 6.013605442 | 0.886516151 | 0.03610225 | 0.023154186 |
| KEGG_PATHWAY | hsa05214:Glioma | 5 | 0.048543689 | 0.009568008 | PRKCB, CDK4, BRAF, RAF1, IGF1R | 98 | 76 | 8840 | 5.934479055 | 0.897565931 | 0.037174064 | 0.023841594 |
| KEGG_PATHWAY | hsa05212:Pancreatic cancer | 5 | 0.048543689 | 0.010009073 | RPS6KB1, CDK4, BRAF, RAF1, VEGFA | 98 | 77 | 8840 | 5.857407898 | 0.907828248 | 0.038260488 | 0.024538372 |
| KEGG_PATHWAY | hsa04140:Autophagy - animal | 7 | 0.067961165 | 0.010542919 | ERN1, RPS6KB1, PDPK1, CTSL, RAF1, CTSD, IGF1R | 98 | 169 | 8840 | 3.736263736 | 0.918889011 | 0.039661458 | 0.025436885 |
| KEGG_PATHWAY | hsa04919:Thyroid hormone signaling pathway | 6 | 0.058252427 | 0.010835536 | RXRA, PDPK1, SRC, PRKCB, RAF1, ESR1 | 98 | 122 | 8840 | 4.43626631 | 0.924380136 | 0.040125344 | 0.025734398 |
| KEGG_PATHWAY | hsa04926:Relaxin signaling pathway | 6 | 0.058252427 | 0.013977264 | MAPK11, MMP13, SRC, NOS3, RAF1, VEGFA | 98 | 130 | 8840 | 4.163265306 | 0.964420303 | 0.050512165 | 0.032395988 |
| KEGG_PATHWAY | hsa05152:Tuberculosis | 7 | 0.067961165 | 0.014066679 | MAPK11, SPHK2, SRC, SPHK1, RAF1, TNF, CTSD | 98 | 180 | 8840 | 3.507936508 | 0.96517685 | 0.050512165 | 0.032395988 |
| KEGG_PATHWAY | hsa04012:ErbB signaling pathway | 5 | 0.048543689 | 0.014580245 | RPS6KB1, SRC, PRKCB, BRAF, RAF1 | 98 | 86 | 8840 | 5.244423351 | 0.969222052 | 0.051574897 | 0.033077571 |
| KEGG_PATHWAY | hsa04910:Insulin signaling pathway | 6 | 0.058252427 | 0.017682661 | PTPN1, RPS6KB1, PDPK1, INSR, BRAF, RAF1 | 98 | 138 | 8840 | 3.921916593 | 0.985422639 | 0.061629275 | 0.039525949 |
| KEGG_PATHWAY | hsa05222:Small cell lung cancer | 5 | 0.048543689 | 0.018922788 | RXRA, CCNE1, CDK4, CDK2, PTGS2 | 98 | 93 | 8840 | 4.849681808 | 0.989194233 | 0.064995664 | 0.041684983 |
| KEGG_PATHWAY | hsa04961:Endocrine and other factor-regulated calcium reabsorption | 4 | 0.038834951 | 0.020198226 | KLK1, PRKCB, ESR1, DNM1 | 98 | 53 | 8840 | 6.807855218 | 0.992061134 | 0.067656879 | 0.043391754 |
| KEGG_PATHWAY | hsa04657:IL-17 signaling pathway | 5 | 0.048543689 | 0.020295691 | MAPK11, HSP90AA1, MMP13, PTGS2, TNF | 98 | 95 | 8840 | 4.747583244 | 0.992246116 | 0.067656879 | 0.043391754 |
| KEGG_PATHWAY | hsa05167:Kaposi sarcoma-associated herpesvirus infection | 7 | 0.067961165 | 0.020553989 | MAPK11, SRC, CDK4, MAPKAPK2, RAF1, PTGS2, VEGFA | 98 | 196 | 8840 | 3.221574344 | 0.992715848 | 0.067656879 | 0.043391754 |
| KEGG_PATHWAY | hsa05169:Epstein-Barr virus infection | 7 | 0.067961165 | 0.023945034 | CCNA2, MAPK11, CCNA1, CCNE1, CDK4, CDK2, TNF | 98 | 203 | 8840 | 3.110485574 | 0.996798133 | 0.077739357 | 0.049858153 |
| KEGG_PATHWAY | hsa05415:Diabetic cardiomyopathy | 7 | 0.067961165 | 0.024979085 | MAPK11, ACE, PARP1, PRKCB, NOS3, INSR, CTSD | 98 | 205 | 8840 | 3.080139373 | 0.997509413 | 0.080000585 | 0.051308392 |
| KEGG_PATHWAY | hsa04921:Oxytocin signaling pathway | 6 | 0.058252427 | 0.026927643 | ROCK1, SRC, PRKCB, NOS3, RAF1, PTGS2 | 98 | 154 | 8840 | 3.514444739 | 0.998449767 | 0.085091353 | 0.054573357 |
| KEGG_PATHWAY | hsa05010:Alzheimer disease | 10 | 0.097087379 | 0.027362155 | ERN1, APP, TUBB2B, TUBA1A, INSR, NOX4, BRAF, RAF1, PTGS2, TNF | 98 | 391 | 8840 | 2.30700976 | 0.998605477 | 0.08532672 | 0.05472431 |
| KEGG_PATHWAY | hsa04730:Long-term depression | 4 | 0.038834951 | 0.0279359 | PRKCB, BRAF, RAF1, IGF1R | 98 | 60 | 8840 | 6.013605442 | 0.998787471 | 0.085841509 | 0.05505447 |
| KEGG_PATHWAY | hsa01100:Metabolic pathways | 26 | 0.252427184 | 0.028251636 | MAOB, ALOX15, HSD17B3, AKR1B1, PTGS2, CYP19A1, PTGS1, CA1, CA2, HSD17B1, HSD17B2, CA4, | 98 | 1561 | 8840 | 1.502438259 | 0.998877323 | 0.085841509 | 0.05505447 |
| KEGG_PATHWAY | hsa04932:Non-alcoholic fatty liver disease | 6 | 0.058252427 | 0.028948481 | ERN1, MAPK11, RXRA, INSR, PPARG, TNF | 98 | 157 | 8840 | 3.447289744 | 0.999052851 | 0.086845443 | 0.055698343 |
| KEGG_PATHWAY | hsa05170:Human immunodeficiency virus 1 infection | 7 | 0.067961165 | 0.029414071 | MAPK11, WEE1, RPS6KB1, PRKCB, CHEK1, RAF1, TNF | 98 | 213 | 8840 | 2.964453387 | 0.999154612 | 0.087139184 | 0.055886734 |
| KEGG_PATHWAY | hsa05160:Hepatitis C | 6 | 0.058252427 | 0.03034786 | RXRA, CDK4, CDK2, BRAF, RAF1, TNF | 98 | 159 | 8840 | 3.403927609 | 0.99932705 | 0.088795589 | 0.05694907 |
| KEGG_PATHWAY | hsa00790:Folate biosynthesis | 3 | 0.029126214 | 0.035009914 | AKR1B10, AKR1C3, AKR1B1 | 98 | 27 | 8840 | 10.02267574 | 0.999785259 | 0.101187191 | 0.064896426 |
| KEGG_PATHWAY | hsa04024:cAMP signaling pathway | 7 | 0.067961165 | 0.03767734 | EDNRA, ROCK1, PTGER3, ADORA1, BRAF, DRD2, RAF1 | 98 | 226 | 8840 | 2.793931752 | 0.999888568 | 0.107584695 | 0.068999467 |
| KEGG_PATHWAY | hsa05022:Pathways of neurodegeneration - multiple diseases | 11 | 0.106796117 | 0.038531466 | ERN1, MAPK11, APP, TUBB2B, TUBA1A, PRKCB, NOX4, BRAF, RAF1, PTGS2, TNF | 98 | 483 | 8840 | 2.054337263 | 0.999909716 | 0.108713779 | 0.069723605 |
| KEGG_PATHWAY | hsa04664:Fc epsilon RI signaling pathway | 4 | 0.038834951 | 0.039882309 | MAPK11, PDPK1, RAF1, TNF | 98 | 69 | 8840 | 5.229222124 | 0.999935301 | 0.111201261 | 0.071318952 |

| | | | | | | | | | | | | |
|---|---|---|---|---|---|---|---|---|---|---|---|---|
| KEGG_PATHWAY | hsa05211:Renal cell carcinoma | 4 | 0.038834951 | 0.041346298 | BRAF, RAF1, MET, VEGFA | 98 | 70 | 8840 | 5.15451895 | 0.999954935 | 0.113632486 | 0.072878219 |
| KEGG_PATHWAY | hsa00591:Linoleic acid metabolism | 3 | 0.029126214 | 0.042482291 | ALOX15, PLA2G2A, PLA2G10 | 98 | 30 | 8840 | 9.020408163 | 0.999965975 | 0.113632486 | 0.072878219 |
| KEGG_PATHWAY | hsa01523:Antifolate resistance | 3 | 0.029126214 | 0.042482291 | ABCC1, TNF, ABCG2 | 98 | 30 | 8840 | 9.020408163 | 0.999965975 | 0.113632486 | 0.072878219 |
| KEGG_PATHWAY | hsa04722:Neurotrophin signaling pathway | 5 | 0.048543689 | 0.042672115 | MAPK11, PDPK1, MAPKAPK2, BRAF, RAF1 | 98 | 120 | 8840 | 3.758503401 | 0.999967537 | 0.113632486 | 0.072878219 |
| KEGG_PATHWAY | hsa04660:T cell receptor signaling pathway | 5 | 0.048543689 | 0.044889199 | MAPK11, PDPK1, CDK4, RAF1, TNF | 98 | 122 | 8840 | 3.696888592 | 0.999981261 | 0.118208224 | 0.075812869 |
| KEGG_PATHWAY | hsa04611:Platelet activation | 5 | 0.048543689 | 0.048334319 | MAPK11, ROCK1, SRC, NOS3, PTGS1 | 98 | 125 | 8840 | 3.608163265 | 0.999992042 | 0.125881688 | 0.080734247 |
| KEGG_PATHWAY | hsa04360:Axon guidance | 6 | 0.058252427 | 0.051474122 | ROCK1, PDPK1, SRC, RAF1, MET, EPHB4 | 98 | 184 | 8840 | 2.941437445 | 0.999996364 | 0.132601813 | 0.085044201 |
| KEGG_PATHWAY | hsa05140:Leishmaniasis | 4 | 0.038834951 | 0.052339483 | MAPK11, PRKCB, PTGS2, TNF | 98 | 77 | 8840 | 4.685926319 | 0.999997071 | 0.133381263 | 0.085544101 |
| KEGG_PATHWAY | hsa04650:Natural killer cell mediated cytotoxicity | 5 | 0.048543689 | 0.054393888 | PRKCB, PRF1, BRAF, RAF1, TNF | 98 | 130 | 8840 | 3.469387755 | 0.999998249 | 0.137142038 | 0.087956075 |
| KEGG_PATHWAY | hsa05202:Transcriptional misregulation in cancer | 6 | 0.058252427 | 0.060764053 | CCNA2, CCNA1, RXRA, PPARG, MET, IGF1R | 98 | 193 | 8840 | 2.804271968 | 0.999999647 | 0.151590322 | 0.097222485 |
| KEGG_PATHWAY | hsa05216:Thyroid cancer | 3 | 0.029126214 | 0.061930128 | RXRA, BRAF, PPARG | 98 | 37 | 8840 | 7.313844457 | 0.999999737 | 0.152890004 | 0.098056036 |
| KEGG_PATHWAY | hsa05130:Pathogenic Escherichia coli infection | 6 | 0.058252427 | 0.072138483 | MAPK11, TUBB2B, TUBA1A, ROCK1, SRC, TNF | 98 | 203 | 8840 | 2.666130492 | 0.99999998 | 0.176255881 | 0.113041747 |
| KEGG_PATHWAY | hsa04976:Bile secretion | 4 | 0.038834951 | 0.076058614 | RXRA, CA2, NR1H4, ABCG2 | 98 | 90 | 8840 | 4.009070295 | 0.999999993 | 0.182079713 | 0.116776863 |
| KEGG_PATHWAY | hsa04211:Longevity regulating pathway | 4 | 0.038834951 | 0.076058614 | RPS6KB1, INSR, PPARG, IGF1R | 98 | 90 | 8840 | 4.009070295 | 0.999999993 | 0.182079713 | 0.116776863 |
| KEGG_PATHWAY | hsa04072:Phospholipase D signaling pathway | 5 | 0.048543689 | 0.080973586 | SPHK2, INSR, SPHK1, RAF1, DNM1 | 98 | 149 | 8840 | 3.026982605 | 0.999999998 | 0.191907399 | 0.123079851 |
| KEGG_PATHWAY | hsa04912:GnRH signaling pathway | 4 | 0.038834951 | 0.082104816 | MAPK11, SRC, PRKCB, RAF1 | 98 | 93 | 8840 | 3.879745447 | 0.999999998 | 0.192661796 | 0.123563684 |
| KEGG_PATHWAY | hsa05323:Rheumatoid arthritis | 4 | 0.038834951 | 0.084165356 | CTSL, CTSK, TNF, VEGFA | 98 | 94 | 8840 | 3.838471559 | 0.999999999 | 0.19556068 | 0.125422883 |
| KEGG_PATHWAY | hsa04930:Type II diabetes mellitus | 3 | 0.029126214 | 0.09377141 | INSR, PRKCE, TNF | 98 | 47 | 8840 | 5.757707338 | 1 | 0.213967219 | 0.137227921 |
| KEGG_PATHWAY | hsa04750:Inflammatory mediator regulation of TRP channels | 4 | 0.038834951 | 0.094795603 | MAPK11, SRC, PRKCB, PRKCE | 98 | 99 | 8840 | 3.644609359 | 1 | 0.213967219 | 0.137227921 |
| KEGG_PATHWAY | hsa05231:Choline metabolism in cancer | 4 | 0.038834951 | 0.094795603 | RPS6KB1, PDPK1, PRKCB, RAF1 | 98 | 99 | 8840 | 3.644609359 | 1 | 0.213967219 | 0.137227921 |

Supplementary Table S2

| Antibody Name | Company | Catalog Number | Protein Molecular | Separation Gel | Transfer Voltage/Curr | ansfer Time (m | Species | Blocking Buffer | king Time ( | Primary Antibody | Primary Antibody | Secondar y | Seconda ry |
|---|---|---|---|---|---|---|---|---|---|---|---|---|---|
| GAPDH | Proteintech Group, Inc | 60004-1-Ig | 36 | 10% | 300mA | 30min | Mouse | 3%BSA | 30min | 1: 10000 | 15h | 1：5000 | 30min |
| VEGFR2 | CST | #2479 | 230 | 8% | 300mA | 50min | Rabbit | 3%BSA | 30min | 1: 1000 | 15h | 1：5000 | 30min |
| P-VEGFR2 | CST | #2478 | 230 | 8% | 300mA | 50min | Rabbit | 3%BSA | 30min | 1: 1000 | 15h | 1：5000 | 30min |
| VEGFA | CST | #50661 | 16/20/23/26 | 12% | 300mA | 20min | Rabbit | 3%BSA | 30min | 1: 1000 | 15h | 1：5000 | 30min |

| **Protein loading amount (determined by BCA concentration measurement to select the loading amount)** | | | |
|---|---|---|---|
| Group | Control | 40μmol/L | 80μmol/L |
| Loading Volume (μl) | 5.0 | 5.0 | 5.0 |

| **Band Density Value (Determined using AlphaEase FC software)** | | | | | | | | | | | | |
|---|---|---|---|---|---|---|---|---|---|---|---|---|
| **Band Intensity Value** | | | | | | | | | | | | |
| Group/Sample ID | GAPDH | VEGFA | P-VEGFR2 | VEGFR2 | GAPDH | VEGFA | P-VEGFR2 | VEGFR2 | GAPDH | VEGFA | P-VEGFR2 | VEGFR2 |
| Control | 961056 | 633888 | 766587 | 654336 | 960160 | 645008 | 768678 | 709488 | 961504 | 633888 | 770608 | 665456 |
| 40μmol/L | 955152 | 555280 | 599808 | 550752 | 953360 | 554368 | 581632 | 605248 | 950624 | 543488 | 595248 | 561632 |
| 80μmol/L | 962427 | 331625 | 447442 | 346125 | 963792 | 346125 | 447988 | 337250 | 963519 | 337250 | 456896 | 342750 |

| The ratio of the grayscale value of the target band to the grayscale value of the internal reference band | | | | | | | | | |
|---|---|---|---|---|---|---|---|---|---|
| | VEGFA | P-VEGFR2 | VEGFR2 | VEGFA | P-VEGFR2 | VEGFR2 | VEGFA | P-VEGFR2 | VEGFR2 |
| Control | 0.66 | 0.80 | 0.68 | 0.67 | 0.80 | 0.74 | 0.66 | 0.80 | 0.69 |
| 40μmol/L | 0.58 | 0.63 | 0.58 | 0.58 | 0.61 | 0.63 | 0.57 | 0.63 | 0.59 |
| 80μmol/L | 0.34 | 0.46 | 0.36 | 0.36 | 0.46 | 0.35 | 0.35 | 0.47 | 0.36 |

| Normalized ratio | | | | | | | | | |
|---|---|---|---|---|---|---|---|---|---|
| | VEGFA | P-VEGFR2 | VEGFR2 | VEGFA | P-VEGFR2 | VEGFR2 | VEGFA | P-VEGFR2 | VEGFR2 |
| Control | 1.00 | 1.00 | 1.00 | 1.00 | 1.00 | 1.00 | 1.00 | 1.00 | 1.00 |
| 40μmol/L | 0.88 | 0.79 | 0.85 | 0.87 | 0.76 | 0.86 | 0.87 | 0.78 | 0.85 |
| 80μmol/L | 0.52 | 0.58 | 0.53 | 0.53 | 0.58 | 0.47 | 0.53 | 0.59 | 0.51 |
